# Global Analysis of mRNA, Translation, and Protein Localization: Local Translation Is a Key Regulator of Cell Protrusions

**DOI:** 10.1016/j.devcel.2015.10.005

**Published:** 2015-11-09

**Authors:** Faraz K. Mardakheh, Angela Paul, Sandra Kümper, Amine Sadok, Hugh Paterson, Afshan Mccarthy, Yinyin Yuan, Christopher J. Marshall

**Affiliations:** 1 Division of Cancer Biology, Institute of Cancer Research, 237 Fulham Road, London SW3 6JB, UK; 2 Division of Molecular Pathology, Institute of Cancer Research, 237 Fulham Road, London SW3 6JB, UK

## Abstract

Polarization of cells into a protrusive front and a retracting cell body is the hallmark of mesenchymal-like cell migration. Many mRNAs are localized to protrusions, but it is unclear to what degree mRNA localization contributes toward protrusion formation. We performed global quantitative analysis of the distributions of mRNAs, proteins, and translation rates between protrusions and the cell body by RNA sequencing (RNA-seq) and quantitative proteomics. Our results reveal local translation as a key determinant of protein localization to protrusions. Accordingly, inhibition of local translation destabilizes protrusions and inhibits mesenchymal-like morphology. Interestingly, many mRNAs localized to protrusions are translationally repressed. Specific *cis*-regulatory elements within mRNA UTRs define whether mRNAs are locally translated or repressed. Finally, RNAi screening of RNA-binding proteins (RBPs) enriched in protrusions revealed *trans-*regulators of localized translation that are functionally important for protrusions. We propose that by deciphering the localized mRNA UTR code, these proteins regulate protrusion stability and mesenchymal-like morphology.

## Highlights

• Local translation is a key regulator of protein localization to cell protrusions
• Localization of mRNAs alone, however, does not determine local protein expression
• Specific UTR elements and RBPs are associated with local translation in protrusions
• Inhibition of local translation destabilizes protrusions

## Teaser

Establishment of front-back cell polarity is the first step in any form of active cell migration. Mardakheh et al. show that in mesenchymal-like cells, front-back asymmetry at the proteome level is maintained by differential local synthesis of cellular proteins.

## Introduction

A pre-requisite of cell migration is the establishment of front-back cell polarity (Etienne-Manneville, 2008). In mesenchymal-like cells, the front is protrusive, characterized by a network of newly generated actin filaments, while the back is retractile, pulling up the body of the cell toward the direction of migration (Sahai and Marshall, 2003). This asymmetry in morphology and function between the front and back has been shown to be associated with differential localization of proteins to protrusions and the cell body, many of which are associated with actin cytoskeleton (Small and Resch, 2005). Actin is polymerized at the leading edge by actin nucleating factors such as the ARP2/3 complex and formins. Other actin binding proteins, such as capping, bundling, and severing proteins, further regulate actin cytoskeleton organization by affecting the dynamics of existing filaments. In the cell body, myosin II association with actin filaments generates contractile force, which is crucial for adhesion maturation and tail retraction (Ridley, 2011). The signaling that initiates protrusions is relayed by a wide variety of signaling molecules. These include lipid second messengers such as phosphatidylinositol-phosphates, as well as members of the Rho family of small GTPases, in particular RHO, RAC, and CDC42, which trigger and coordinate front-back polarization and spatial organization of the actin cytoskeleton (Cain and Ridley, 2009, Ridley, 2011).

While much is known about the mechanisms that trigger protrusion formation, little is known about how front-back cell asymmetry is maintained in migrating cells. In addition to differential distribution of proteins, mRNAs are also differentially distributed between the front and back of the cells (Condeelis and Singer, 2005, Mili et al., 2008, Mingle et al., 2005). Cellular mRNAs are known to be transported via microtubule or actin filaments (St Johnston, 2005). Once localized, mRNAs could be subject to local translation (Halstead et al., 2015, Hüttelmaier et al., 2005, Yasuda et al., 2013), which would result in increased local concentration of their corresponding proteins. Importantly, the mechanisms that regulate mRNA targeting and translation in protrusions are poorly defined. Moreover, to what extent mRNA localization and local translation define front-back asymmetry and protrusion formation remains to be determined.

Here, we assessed how mRNA localization and local translation contributes toward front-back asymmetry at the proteome level. We used a proteomics approach, quantifying the distribution of cellular proteins between protrusions and the cell body by stable isotope labeling of amino acids in culture (SILAC) (Ong et al., 2002). Using a pulse labeling variation of the SILAC strategy known as pulsed-SILAC (Selbach et al., 2008), we also determined the relative translation rates of proteins between protrusions and the cell body. Finally, we quantified the relative distribution of mRNAs between protrusions and the cell body by RNA sequencing (RNA-seq). Comparison of relative protein distributions with mRNA distributions showed little correlation between the two, suggesting that mRNA localization alone does not determine protein localization to protrusions. However, relative translation rates between protrusions and the cell body exhibited a significant correlation with protein distributions, suggesting that localized translation is a significant determinant of front-back cell asymmetry. Accordingly, we show that local protein translation in protrusion is crucial for stabilizing protrusions and maintaining a mesenchymal-like polarized morphology. Analysis of mRNA UTR elements revealed several conserved motifs that were enriched in locally translated mRNAs, suggesting that their absence or presence can control local translation rates. To reveal *trans*-regulators of local translation that were functionally important for protrusions, we screened RBP categories enriched in protrusions by RNAi. We found several RBPs that significantly regulated protrusions and mesenchymal-like migration. These included many known but also a number of novel regulators of mRNA localization and local translation including the exosome core complex (Lykke-Andersen et al., 2009). We demonstrate that the exosome core, but not its catalytic subunits, is highly enriched in protrusions and is essential for protrusion stability but not initiation. Our work establishes local translation as a key regulator of asymmetric distribution of the proteome between protrusions and the cell body, describes *cis*-regulatory UTR motifs that are associated with localized translation in protrusions, and highlights *trans*-acting RBPs that are involved in regulation of protrusions and mesenchymal-like migration.

## Results

### Local Translation Is a Key Determinant of Protein Localization to Protrusions

**Figure 1:**
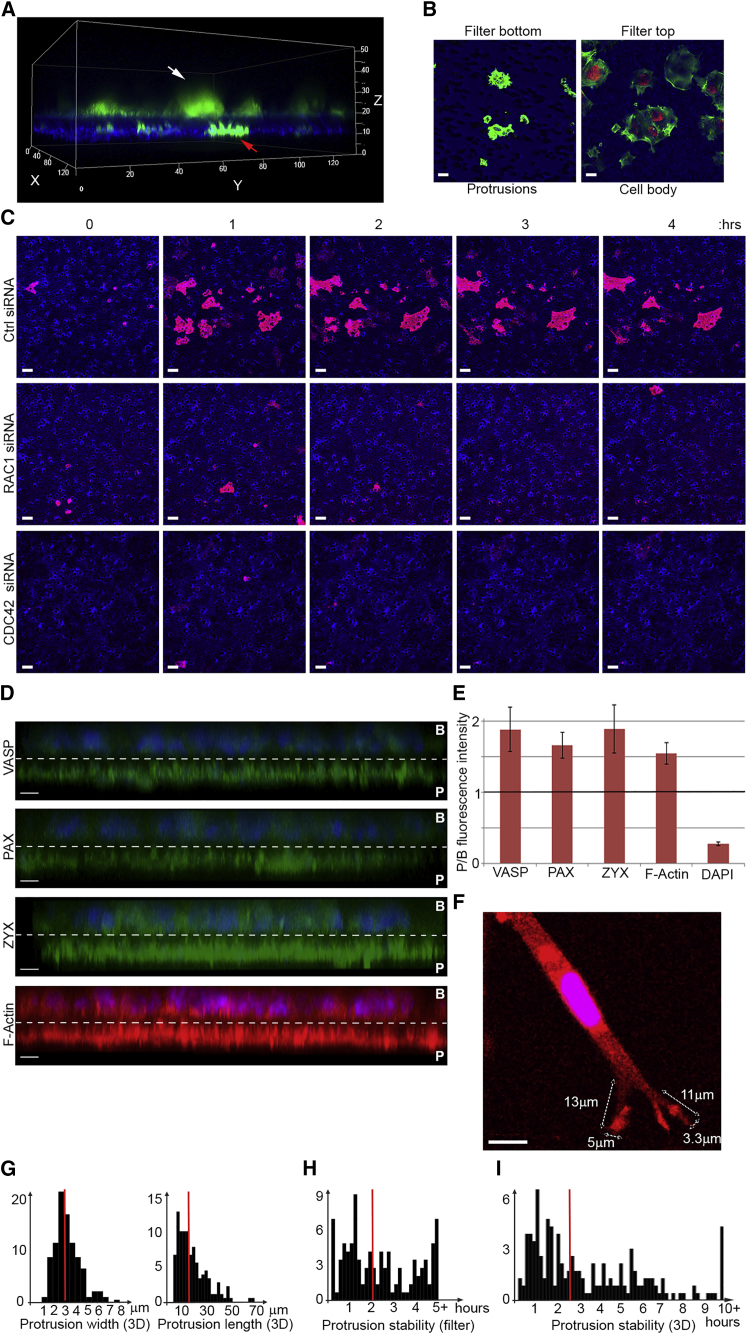
Filter-Based Analysis of Protrusions in MDA-MB231 Cells (A) Confocal 3D rendering of MDA-MB231 cells forming protrusions through 3-μm transwell filters. Cells were stained with CellTracker-CMFDA (green) 24 hr prior to seeding. The collagen coated filter was stained prior to seeding by CellTrace Far Red DDAO-SE (blue). White arrow, cell body; red arrow, protrusions. Axes scales are in μm. (B) Transwell separated protrusions are actin-rich, but lack a nucleus. MDA-MB231 cells protruding through 3-μm transwell filters were fixed and stained with phalloidin-Alexa Fluor-488 (green) and Hoechst (red). Confocal images were taken from the top (right image) and bottom (left image) of the filter. The filter was visualized by transmitted light (blue). Scale bar, 10 μm. (C) Formation of protrusions is dependent on RAC1 and CDC42. Control, RAC1, or CDC42 siRNA transfected MDA-MB231 mKate-CAAX cells (red) were seeded on collagen-coated 3-μm transwell filters and time-lapsed as they formed protrusions through the pores. Confocal images were taken from the bottom of the filter. Collagen coated filters were stained by CellTrace Far Red DDAO-SE (blue) prior to seeding. Scale bar, 10 μm. (D) Protrusions formed through transwell filters are enriched in VASP, PXN, ZYX, and F-actin. Cross-section side views of confocal 3D renderings of MDA-MB231 cells protruding through 3-μm transwell filters. Cells were fixed and immuno-stained with antibodies against indicated markers (green), or phalloidin for F-actin staining (red). DAPI was used for nuclear staining (blue). Dashed line marks the position of the filter. Protrusions are marked as (P) and the cell-bodies are marked as (B). Scale bar, 10 μm. (E) Quantification of the protrusion (P)/cell body (B) fluorescent intensities for indicated markers from (D). At least three independent tiled scans, each formed from nine fields of view, were used. All error bars are SD. (F) Protrusion diameters of MDA-MB231 cells in 3D pepsinized collagen-I are comparable to transwell protrusion diameters. MDA-MB231 mKate-CAAX cells (red) were seeded on 3D collagen-I gels and imaged 2–4 hr after seeding by confocal microscopy. Scale bar, 10 μm. (G) Protrusion width and length measurements from 92 cells (as in F) are displayed as histograms. A total of 172 individual protrusions were measured. Average protrusion width in 3D collagen-I is 3.1 μm (red line). Average protrusion length in 3D collagen-I is 16.5 μm (red line). (H) Transwell protrusions are stable for a variety of time lengths, with a median stability of 2 hr and 5 min. The stability of protrusions was assessed from nine time-lapse movies of protrusion formation through 3-μm transwell filters (see also Movie S1). A total of 136 protrusions from 85 cells were timed over a 5-hr period, and the results are displayed as a histogram. Red line marks the median stability (125 min). (I) 3D protrusions are stable for a variety of time-lengths, with a median stability of 2 hr and 42 min. The stability of protrusions was assessed from four time-lapse movies of protrusion formation in 3D collagen-I (see also Movie S2). A total of 200 protrusions from 99 cells were timed over a 10-hr period. The results are displayed as a histogram. Red line marks the median stability (162 min).

To study cell protrusions, we used a micro-porous filter-based method to separate protrusions from cell bodies of highly protrusive MDA-MB231 breast cancer cells (Shankar et al., 2010, Wang et al., 2007). In this method, cells are seeded on top of collagen-I-coated 3-μm transwell filters to enable protrusions to form but to prevent the cell bodies from passing through due to the size of the pores, resulting in separation of protrusions and the cell bodies on opposite sides of the filter (Figures 1A and 1B). As expected (Sanz-Moreno et al., 2008), these protrusions are dependent on Rho-GTPases RAC1 and CDC42 (Figure 1C) and are enriched in known protein markers of protrusive fronts such as VASP (Rottner et al., 1999), PAX (Nobes and Hall, 1995), ZYX (Crawford et al., 1992), as well as F-actin (Figures 1D and 1E). In terms of spatial and temporal dynamics, protrusion formation through transwell pores seems to be a good mimic of protrusion formation in 3D matrices, where cells have to similarly protrude through matrix pores and holes. In fact, the transwell pore size of 3 μm is comparable to the average pore size of pepsinized collagen-I 3D matrices (Wolf et al., 2009), and average diameters of protrusions extended through these transwell pores (∼3 μm wide, ∼10–15 μm long, dictated by pore size diameters) are comparable to the diameters of protrusions in 3D collagen-I (Figures 1F and 1G). In addition, the temporal dynamics of protrusions extended through transwell pores are similar to those extended in 3D collagen-I, with protrusions remaining stable for various lengths of time, from minutes to several hours, with a median length of over 2 hr (Figures 1H and 1I; Movies S1 and S2).

**Figure 2:**
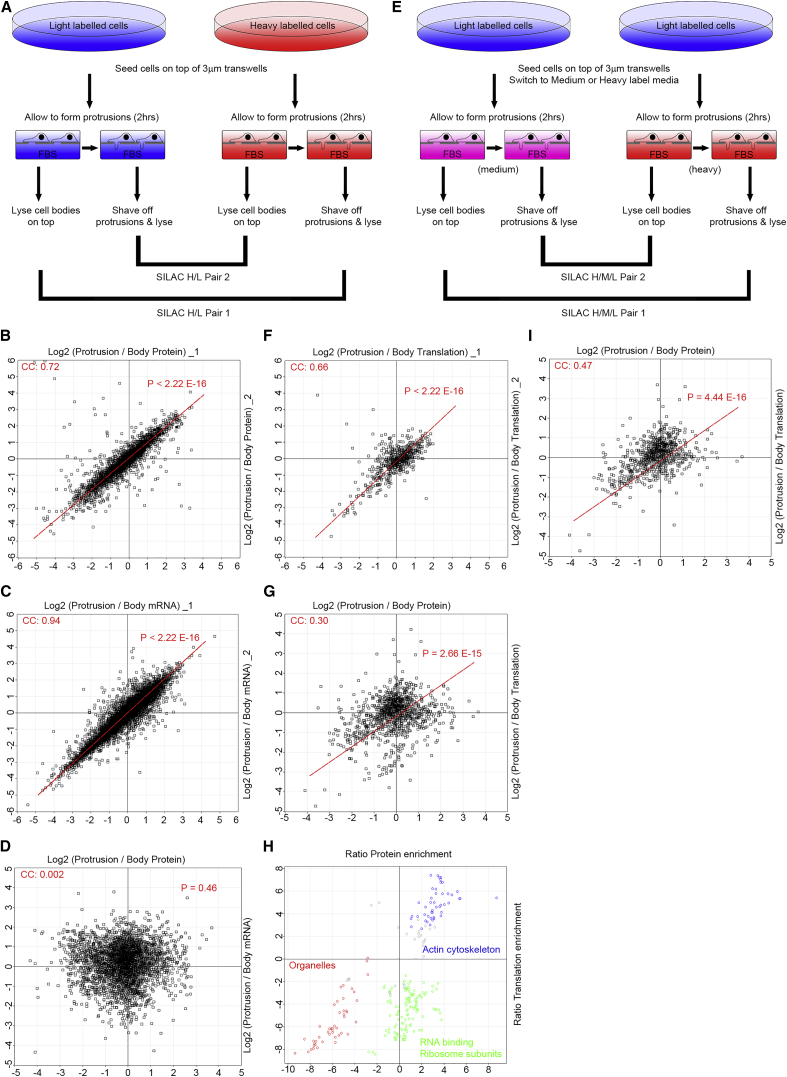
Localized Translation, but Not mRNA Targeting, Significantly Contributes toward the Proteome Asymmetry between Protrusions and the Cell Body (A) Schematic representation of SILAC protrusion proteomics analysis; the H/L ratios are the measures of protrusion/body protein distributions. (B) Proteome distribution between protrusions and the cell body. Log2 SILAC ratios from reciprocal SILAC mixtures of protrusion and cell body fractions (Data S1A) were plotted against each other. CC, Pearson’s correlation coefficient between the two pairs. P value of the correlation is displayed on the graph. (C) Messenger-RNA distributions between protrusions and the cell body. Log2 of protrusion/cell body FPKM ratios from two replicate experiments (Data S1C) were plotted against each other. CC, Pearson’s correlation coefficient between the two replicates. P value of the correlation is displayed on the graph. (D) Protein distributions between protrusions and the cell body do not correlate with mRNA distributions. Averaged Log2 of protrusion/cell body protein ratios were plotted against averaged Log2 of Protrusion/Cell body mRNA ratios. CC = Pearson’s correlation coefficient. P-value of the correlation is displayed on the graph. (E) Schematic representation of pSILAC protrusion proteomics analysis. H/M ratios for each protein are the measures of protrusion/body translation rates. (F) Translation rate distributions between protrusions and the cell body. Log2 of H/M SILAC ratios from reciprocal pSILAC mixtures of protrusion and cell body fractions (Data S1D) were plotted against each other. CC, Pearson’s correlation coefficient between the two pairs. P value of the correlation is displayed on the graph. (G) Protein distributions between protrusions and the cell body significantly correlate with translation rate distributions. Averaged Log2 of protrusion/cell body protein ratios from the two reciprocal SILAC pairs were plotted against averaged Log2 of protrusion/cell body pSILAC ratios. CC, Pearson’s correlation coefficient. P value of the correlation is displayed on the graph. (H) RNA-binding proteins and ribosomal components are enriched but not locally synthesized in protrusions. The 2D-annotation enrichment analysis data from Data S1E was plotted with each data point representing a protein category. Most protein categories exhibit a correlative regulation of their protein distributions and relative translation rates, for example all protrusion-enriched actin-related categories (blue), and all protrusion-depleted organelle-related categories (red), but an anti-correlative behavior is observed for RNA-binding and ribosomal protein categories (green), with their relative translation rates significantly lower than their relative protein amounts. (I) Removal of RNA-binding and ribosomal protein categories from (G) significantly increases the correlation between protein distributions and translation rates from 0.30 to 0.47. CC, Pearson’s correlation coefficient. P value of the correlation is displayed on the graph. See also Figure S1.

Using the transwell-based fractionation method, we first determined the distribution of cellular proteins between protrusions and the cell body by quantitative proteomics (Figure 2A), revealing the relative distribution of 3,334 proteins from two reciprocally labeled SILAC experiments (Data S1A). Overall, the proteome exhibited a normal-like distribution between protrusions and the cell body, with different proteins being enriched or depleted in protrusions to various levels (Figure 2B). Annotation enrichment analysis (Cox and Mann, 2012) revealed multiple protein categories that were significantly enriched or depleted in protrusions (Data S1B). As expected, the majority of the protrusion-enriched proteins belonged to known actin-associated categories (Data S1B). These included upstream signaling regulators of protrusion formation, actin polymerizing factors such as the ARP2/3 complex and its upstream regulator WAVE2, as well as other actin binding and adhesion molecules such as VASP, ENAH, and PXN (Data S1B). On the other hand, the majority of cell body-enriched proteins were those of nucleus, ER, mitochondria, and other organelles (Data S1B). Interestingly, several categories of RNA-binding proteins (RBPs), translation factors, and protein folding chaperones were enriched in protrusions (Data S1B), indicating that a significant level of localized protein synthesis may occur in protrusions.

In order to determine to what level mRNA localization contributes toward protein localization in protrusions, we quantified the distribution of cellular mRNAs between protrusions and the cell body by RNA-seq (Figure S1A; Data S1C). Similar to the proteome distribution, RNA-seq revealed a normal-like distribution, with different mRNAs being enriched or depleted to different degrees between protrusions and the cell body (Figure 2C). Surprisingly, no correlation was detected between mRNA and protein distributions (Figure 2D), suggesting that mRNA localization alone is not a significant predictor of protein localization to protrusions. As a validation of our method, we compared our RNA-seq dataset against a previously published microarray-based protrusion/cell body mRNA localization study in mouse NIH/3T3 fibroblasts (Mili et al., 2008). There was a very strong overlap between the two datasets (Figure S1B), suggesting that RNA localization at the global level is likely to be conserved across species and cell types, despite a lack of correlation with protein distributions.

Next, we assessed whether local translation contributes toward protein localization to protrusions. We quantified relative translation rates of proteins between protrusions and the cell body during a 2 hr transwell protrusion formation assay by utilizing a variation of the SILAC method called pulsed SILAC (pSILAC) (Selbach et al., 2008) (Figure 2E). We determined the relative translation rates of 1,150 proteins between protrusions and the cell body (Data S1D). The lower coverage compared to the proteome analysis was expected, as the medium and heavy peptides that are used for pSILAC quantification are significantly low in abundance after a short 2 hr labeling. Nevertheless, widespread localized translation was evident, with many cellular proteins displaying higher translation in protrusions than the cell body and vice-versa (Figure 2F). Moreover, we observed a significant correlation between relative translation rate and protein localization (Figure 2G). Pearson’s correlation coefficient between protein and translation rate distributions was 0.30. The actual correlation is likely to be even higher, considering that the Pearson’s correlation coefficients between the two SILAC and pSILAC replicates were 0.72 and 0.66, respectively, presumably due to the inherent errors of the mass spectrometry-based measurements (Figures 2B and 2F). These results demonstrate that localized translation is a key determinant of a significant degree of protein localization to protrusions.

### Ribosomes and RNA-Binding Proteins Are Not Locally Translated but Are Transported to Protrusions

We next investigated if the contribution of local translation toward protein localization was uniform across all protein categories or whether certain categories of proteins were more reliant on local translation than others for their localization. We performed a 2D annotation enrichment analysis (Cox and Mann, 2012) to determine which categories of proteins show a positive correlation between their protein enrichment levels and their relative translation rates as opposed to those that exhibit a negative correlation (Data S1E). All actin-associated protrusion-enriched categories showed a strong positive correlation, having both high protrusion protein levels and high protrusion translation levels (Figure 2H). These categories included ARP2/3 complex subunits, adhesion-related proteins, ezrin, filamins, alpha-actinin, and other actin-binding protrusion proteins such as actin capping and LIM domain proteins (Data S1E). Most organelle-associated protein categories depleted in protrusions also showed a positive correlation, exhibiting both low protrusion protein and low protrusion translation levels (Figure 2H). Collectively, these results suggest that the localization of these protein categories is likely to be determined by their localized translation, either in protrusions or at the cell body. In contrast, RNA-binding and ribosomal protein categories exhibited a negative correlation, having significantly lower protrusion translation rates compared to their protrusion protein levels (Figure 2H). In fact, removal of ribosomal and RNA-binding proteins from our datasets resulted in a significant increase in the overall correlation from 0.30 to 0.47 (Figure 2I). These results suggest that while the localization of most protein categories, including all actin-associated protrusion proteins, can be explained by the differences in their localized translation rates, RNA-binding and ribosomal proteins do not follow this rule, indicating that these proteins are likely to be transported to protrusions instead.

### Local Translation Is Needed for Protrusion Stability

**Figure 3:**
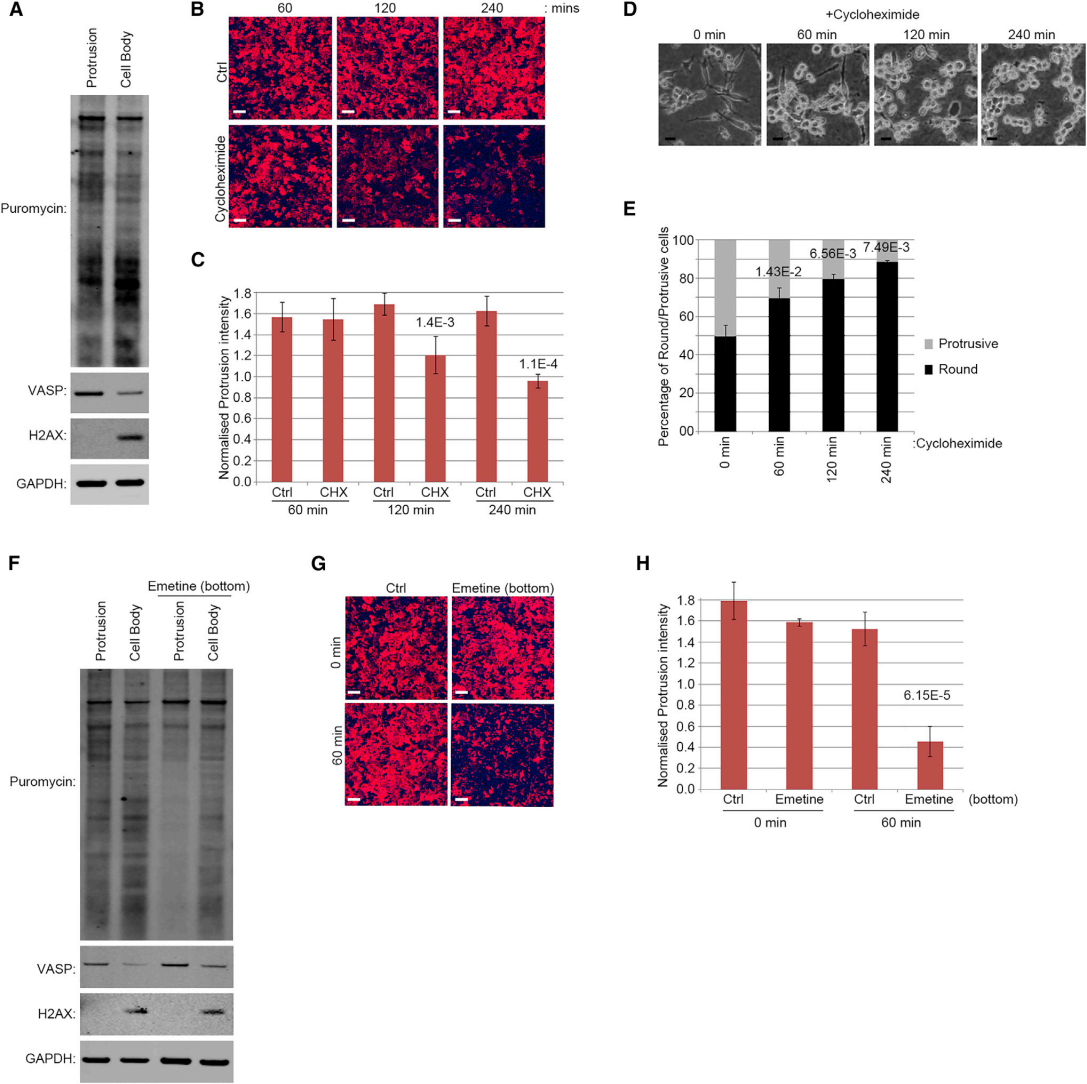
Local Translation Is Needed for Protrusion Stabilization (A) Widespread translation occurs in protrusions. MDA-MB231 cells seeded on 3-μm transwells for 2 hr were treated with Puromycin (10 μg/ml) for 10 min to label nascent proteins. Equal amounts of isolated protrusion and cell body fractions were then resolved by SDS-PAGE and blotted for puromycinylation, VASP as a protrusion marker, H2AX as a cell body marker, and GAPDH as loading control. (B) Protrusions initiate but retract in translation-inhibited cells. MDA-MB231 mKate-CAAX cells were seeded on 3-μm transwells in presence or absence of cycloheximide (10 μg/ml), fixed at indicated times, and analyzed by confocal microscopy. Images show protrusions at the bottom of transwell filters. Red, cell membranes; blue, filter. Scale bar, 50 μm. See also Movie S3. (C) Quantification of protrusions from (B) (n = 4). The significant p values are stated above the bar graph. Error bars are SD. (D) Translation inhibition switches cell morphology from protrusive to round in 3D. MDA-MB231 cells were seeded on top of 3D collagen-I gels for 4 hr and treated with cycloheximide (10 μg/ml) for indicated times before being fixed and imaged. Scale bar, 50 μm. See also Movie S4. (E) Quantification of protrusive versus round morphologies from (D) (n = 3). Significant p values are stated above each bar graph. Error bars are SD. (F) Local inhibition of translation in protrusions. MDA-MB231 cells were seeded on 3-μm transwells for 2 hr. Emetine (1 μg/ml) was then added to the bottom chamber for 5 min as in Figure S2F. Transwells were then washed and treated with Puromycin (10 μg/ml) for 10 min to label nascent proteins before lysis. Equal amounts of isolated protrusion and cell body fractions were then resolved by SDS-PAGE and blotted for puromycinylation, VASP (protrusion marker), H2AX (cell body marker), and GAPDH (loading control). The emetine treatment specifically inhibits translation in protrusions. (G) Inhibition of translation in protrusions destabilizes protrusions. MDA-MB231 mKate-CAAX cells were seeded on 3-μm transwells for 2 hr before being treated with emetine (1 μg/ml) or vehicle as in (F). The cells were then either fixed immediately (0 min), or left for 1 hr (60 min) before being fixed and analyzed by confocal microscopy. The images show protrusions at the bottom of the filter. Red, cell membranes; blue, filter. Scale bar, 50 μm. See also Movie S5. (H) Quantification of protrusions from (G) (n = 10). The significant p values are stated above the bar graph. Error bars are SD. See also Figure S2.

Our pSILAC data suggest a significant role for local translation in regulation of the asymmetry between protrusions and the cell body at the proteome level. Thus, we predicted that inhibition of local translation in protrusion should inhibit protrusions. We first confirmed the occurrence of localized translation in protrusions by visualizing nascent protein synthesis in protrusion and cell body fractions, using puromycinylation tagging of newly synthesized proteins (Schmidt et al., 2009). Puromycinylation analysis of protrusion and cell body fractions confirmed that protein translation was widespread in protrusions, with overall levels comparable to that of the cell body (Figure 3A). High levels of translation in protrusions could also be revealed by immunofluorescence staining of transwell protruding cells using anti-puromycin staining following puromycinylation (Figure S2A).

Next, we tested whether protein translation was needed for protrusion formation. Treatment of MDA-MB231 cells with cycloheximide-abrogated protein translation (Figures S2A and S2B). Temporal analysis of protrusion formation revealed that MDA-MB231 cells still initiated protrusions in presence of cycloheximide, but these protrusions were unstable and retracted back (Figures 3B and 3C; Movie S3), suggesting that translation acts to stabilize protrusions rather than to initiate them. Cycloheximide treatment also destabilized protrusions in a 3D collagen-I matrix, resulting in conversion of the cells from a polarized mesenchymal-like morphology to round (Figures 3D and 3E; Movie S4). The rounding observed with cycloheximide treatment was not due to apoptosis (Figure S2C), suggesting that protein translation is needed for protrusion stability.

To show that it is localized translation that is needed for protrusion stability, we devised a strategy to inhibit translation specifically in protrusions. We used emetine, an irreversible inhibitor of protein translation. Emetine concentrations as low as 1 μg/ml, and treatment times as short as 5 min could strongly abrogate translation in MDA-MB231 cells (Figures S2D and S2E). To locally inhibit translation in protrusions, a low dose of 1 μg/ml emetine was added to the bottom of transwell filters for 5 min. In order to limit emetine diffusion upward, a positive hydrostatic pressure was generated to push liquid down from the top to the bottom of the filter (Figures S2F and S2G), allowing inhibition of translation in protrusions while leaving the cell-bodies unaffected (Figures 3F and S2H). Importantly, this local inhibition of translation also destabilized protrusions (Figures 3G and 3H; Movie S5), demonstrating that local translation is critical for the stability of protrusions.

### Specific 3′UTR Motifs Are Associated with Local Translation

**Figure 4:**
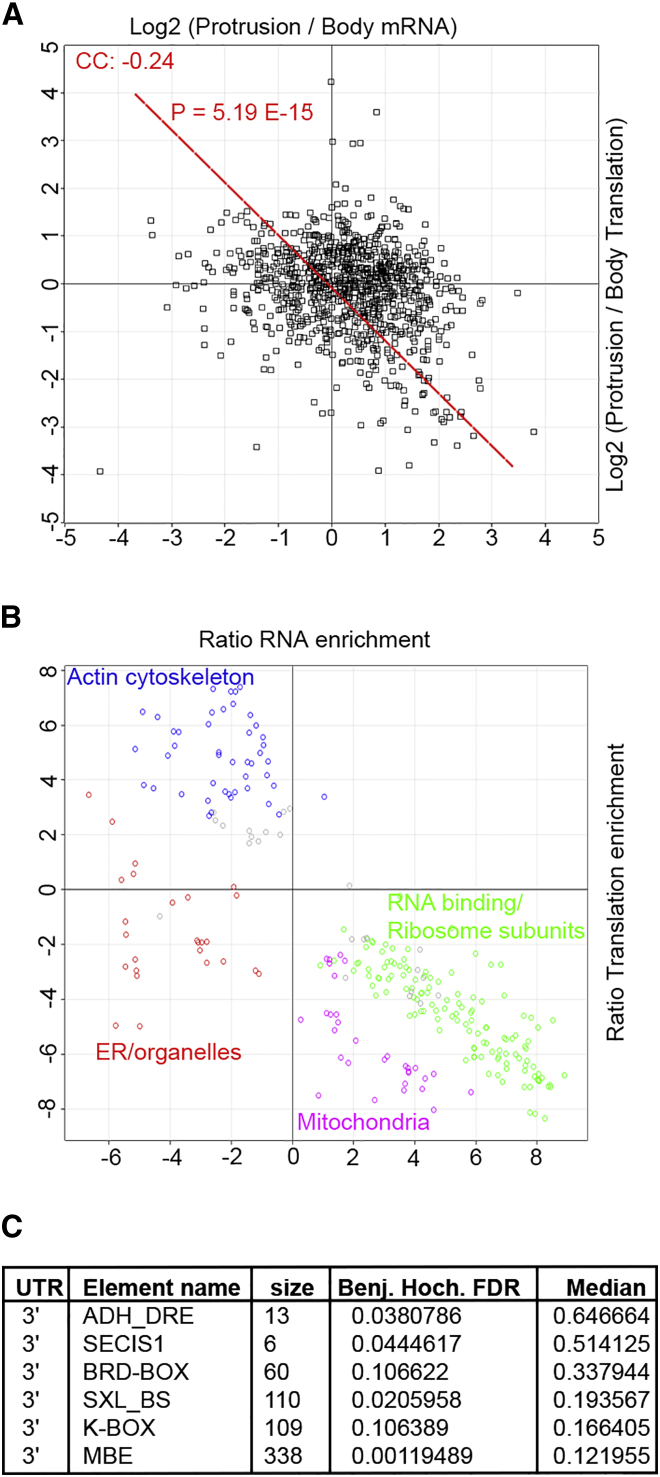
mRNA Enrichment Does Not Determine Local Translation Rates in Protrusions (A) mRNA distributions between protrusions and the cell body negatively correlate with translation rate distributions. Averaged Log2 of protrusion/cell body mRNA FPKM ratios from two replicate experiments were plotted against averaged Log2 of protrusion/cell body pSILAC translation ratios. CC, Pearson’s correlation coefficient. P value of the correlation is displayed on the graph. (B) Most categories of proteins show a negative correlation between their mRNA localization and local translation. The 2D-annotation enrichment analysis data from Data S2A was plotted with each data point representing a protein category. Most actin cytoskeleton-related categories (blue) show high relative local translation rates but their mRNA is not highly enriched in protrusions while mitochondrial (purple) and RNA-binding/ribosomal (green) protein categories show high relative mRNA enrichment but low local translation rates in protrusions. mRNAs for ER and other organelle categories (red) are enriched and translated more in the cell body. (C) Specific 3′ UTR elements are associated with higher local translation rates in protrusions. Data of averaged Log2 of protrusion/cell body pSILAC translation ratios were annotated for known UTR elements from UTRdb (Grillo et al., 2010) (see also Data S2B), and subjected to 1D annotation enrichment analysis. Six UTR elements were significantly enriched in locally translated mRNAs as listed. Size, the number of mRNAs annotated with each element in the dataset; Benj. Hoch. FDR, Benjamini Hochberg false detection rate for enrichments; median, median of the averaged Log2(protrusion/cell body) pSILAC translation ratios for each UTR category.

Despite the presence of mRNA in protrusions being a logical pre-requisite for any localized translation, our results showed little correlation between mRNA and protein distributions. We hypothesized that this might be due to some protrusion-localized mRNAs remaining translationally inactive in protrusions. In support of this, mRNA and relative translation rate distributions between protrusions and the cell body negatively correlate with each other (Figure 4A). Furthermore, 2D annotation enrichment analysis revealed that most protein categories also show a negative correlation between their mRNA and translation distributions (Figure 4B), with actin cytoskeleton-associated protein categories having relatively high protrusion translation levels but low mRNA enrichments, while mitochondrial, as well as RNA-binding and ribosomal proteins have high relative protrusion mRNA levels but low relative translation in protrusions (Figure 4B; Data S2A). Collectively, these results suggest that for certain categories of mRNAs, targeting to protrusion may serve as a mechanism for translation repression, while for others, low-level mRNA presence in protrusion maybe enough to support robust local translation.

As mRNA enrichment in protrusions does not define local translation rates, we next set out to reveal the properties in mRNA sequence that may be governing whether an mRNA is locally translated or repressed in protrusions. *Cis*-regulatory elements in the 5′ and 3′ UTRs of mRNAs play a pivotal role in regulation of translation (Grillo et al., 2010). Thus, we investigated whether presence or absence of specific UTR motifs could be-associated with differences local translation rates. We focused on 58 well-known 5′ and 3′ UTR regulatory elements (Grillo et al., 2010) (Data S2B) and asked whether presence or absence of any of these elements was-associated with local translation in protrusions. We found six elements that were significantly-associated with higher local translation in protrusions (Figure 4C). The best characterized of these elements is Musashi binding element (MBE), the binding element for MSI1, which can act as both activator and inhibitor of translation depending on the context (Charlesworth et al., 2006). Interestingly, MSI1 has recently been demonstrated to bind to and regulate ARP2/3 subunits mRNAs in neuronal cells (Hadziselimovic et al., 2014), a role in agreement with its involvement in protrusions. Existence of an association between specific UTR motifs and local translation rates suggests that such motifs are likely to be locally controlling translation in protrusions.

### KH, PH, and Sm Domain-Containing RBPs Modulate Protrusions

**Figure 5:**
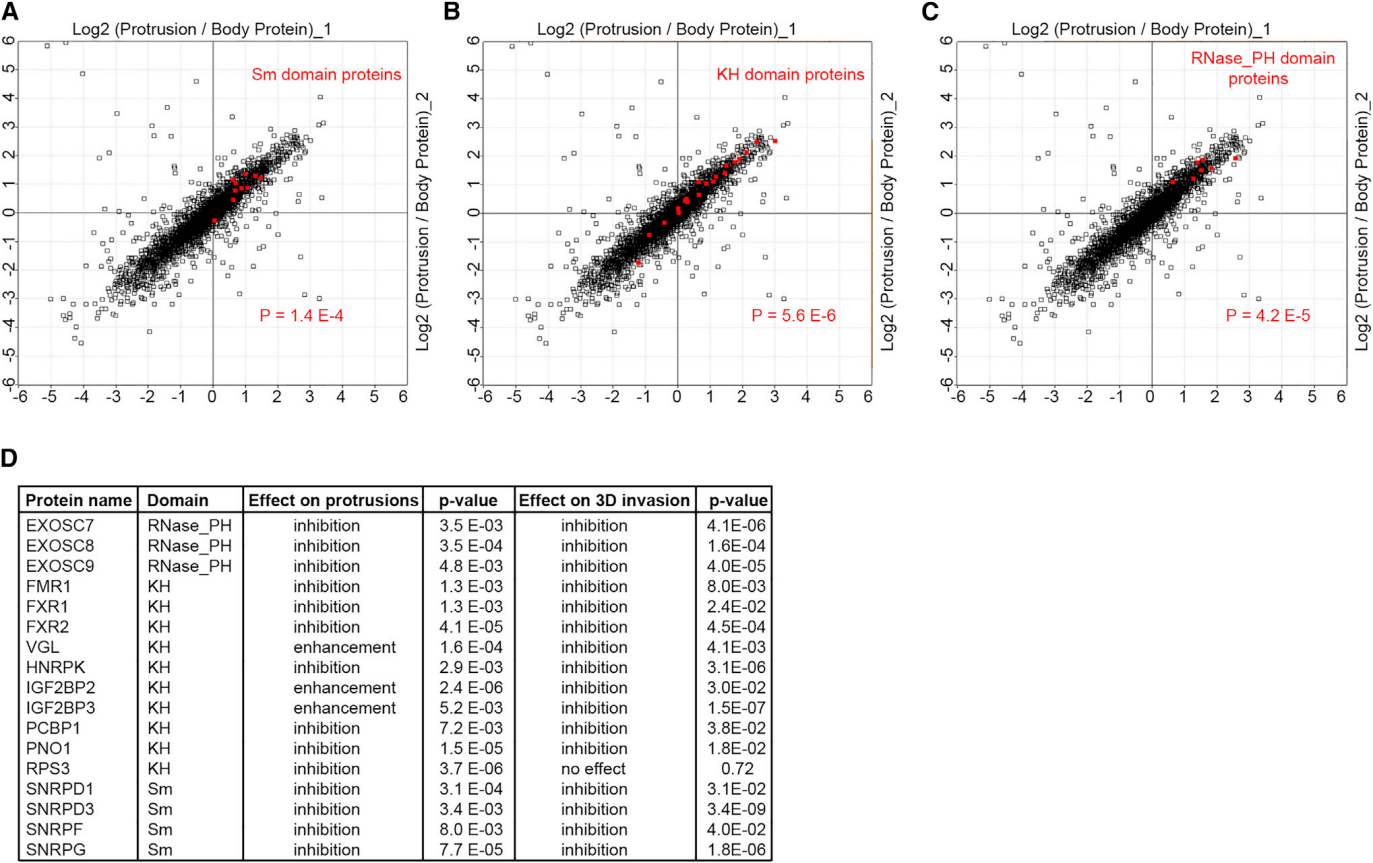
Specific Categories of RBPs Are Enriched in and Functionally Important for Protrusions (A) Sm domain-containing RBPs are significantly enriched in protrusions. Log2 of SILAC-quantified protein ratios were plotted against each other with all Sm domain proteins marked in red. P value for the annotation enrichment is displayed on the graph. (B) KH domain-containing RBPs are significantly enriched in protrusions. Log2 of SILAC-quantified protein ratios were plotted against each other with all KH domain proteins marked in red. P value for the annotation enrichment is displayed on the graph. (C) RNase PH domain-containing RBPs are significantly enriched in protrusions. Log2 of SILAC-quantified protein ratios were plotted against each other with all RNase PH domain proteins marked in red. P value for the annotation enrichment is displayed on the graph. (D) List of all significant hits from RNAi screening of protrusion-enriched RBPs in MDA-MB231 mKate-CAAX cells, their depletion effect on protrusions (inhibition or enhancement), as well as their depletion effect on 3D invasion into collagen-I. First p value column is for the t test significance of the change in protrusions. Second p value column is for the t test significance of the change in 3D invasion index.

As translation is mainly governed by different RBPs and translation factors that act upon an mRNA, RBPs that are localized to protrusions are likely to be crucial for local regulation of translation. Thus, to reveal RBPs that are functionally important for protrusions, we performed an RNAi screen for protrusion-enriched RBPs. Category enrichment analysis revealed three families of RBPs that were significantly enriched in protrusions (Data S1B). These were Sm, KH, and RNase PH domain-containing proteins (Figures 5A–5C). As enrichment of multiple structurally related proteins can be indicative of a conserved function, we focused on the members of these categories, depleting each protein by RNAi and assessing the ability of depleted cells to form stable protrusions through transwell filters. Out of a total of 38 RBPs screened, knockdown of 17 significantly impacted on protrusions (Figure 5D). Interestingly, while the majority of knockdowns inhibited protrusions, some enhanced them (Figure 5D), suggesting that different protrusion localized RBPs function in opposite manners to fine-tune protrusion dynamics.

To further validate the role of these RBPs in mesenchymal-like migration, we assessed the invasion of MDA-MB231 cells through a 3D collagen-I matrix, where they preferentially migrate as mesenchymal-like cells, following siRNA depletion. Strikingly, knockdown of 16 out of the 17 RBP hits significantly impaired the invasive potential of MDA-MB231 cells (Figure 5D), suggesting that most of these RBPs are likely to play a role in regulation of mesenchymal-like cell migration in 3D. Interestingly, RBP knockdown of both negative as well as positive regulators of protrusions had a negative impact on the invasive potential of the cells (Figure 5D), suggesting that a precise fine tuning of protrusion formation is crucial for optimal migration in 3D.

Several of the identified RBPs such as the fragile X mental retardation protein-1 (FMR1) and its two homologs (FXR1 and FXR2), zipcode-binding proteins (IGF2BP2 and IGF2BP3), heterogeneous nuclear ribonucleoprotein K (HNRPK), and small nuclear ribonucleoprotein core subunits (SNRPD1, SNRPD3, SNRPF, SNRPG), have been reported to regulate mRNA localization or local translation (Boudoukha et al., 2010, Comery et al., 1997, Gu et al., 2012, Liu et al., 2008, Nielsen et al., 2001, Schaeffer et al., 2012, Taniuchi et al., 2014, Vasudevan and Steitz, 2007, Zalfa et al., 2006, Zhang et al., 1995). Others such as vigilin (VGL), partner of NOB1 (PNO1), and ribosomal protein subunit-3 (RPS3), either associate with, or are part of the translation machinery itself (Kim et al., 2010, Kruse et al., 2003, Vanrobays et al., 2004). However, a number of the identified proteins were not previously implicated in regulation of local translation, including three subunits of the exosome core complex (EXOSC7, EXOSC8, and EXOSC9), an RNA degrading complex involved in maturation, quality control, and turnover of many types of cellular RNA (Lykke-Andersen et al., 2009) (Figure 5D), suggesting that they are likely to be novel regulators of local translation in protrusions.

### The Exosome Complex Is a Regulator of Cell Protrusions, Morphology, and Migration

**Figure 6:**
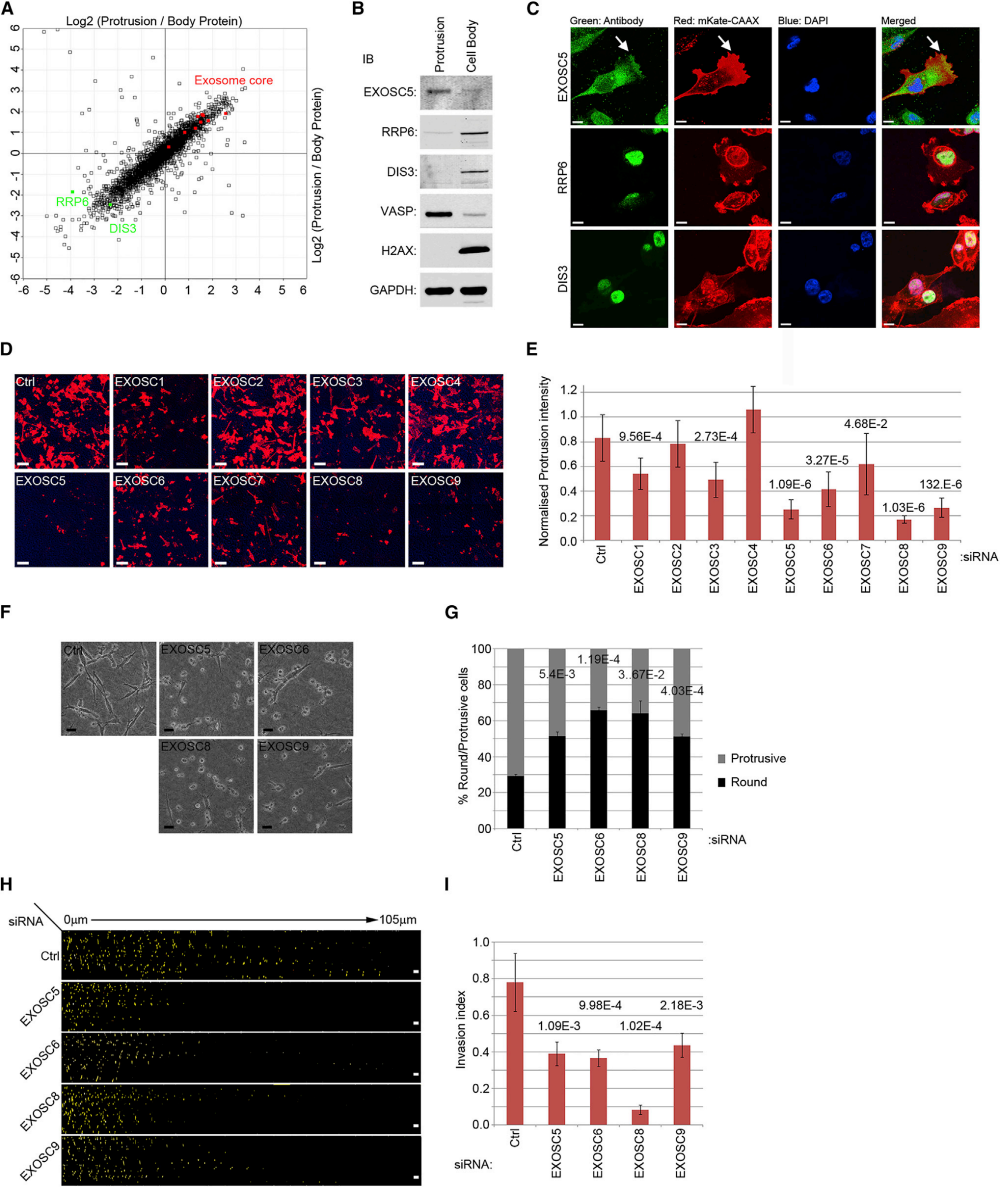
Exosome Core Is Enriched in Protrusions and Is Required for Protrusion Stability, Mesenchymal-like Morphology, and 3D Migration (A) Exosome core subunits, but not catalytic subunits, are enriched in protrusions. Log2 of SILAC-quantified protein ratios were plotted against each other with exosome core subunits marked in red and catalytic subunits in green. All nine exosome core subunits were present more in protrusions than the cell body with eight showing at least 2-fold enrichment, while both expressed catalytic subunits show a strong enrichment in the cell body. (B) Western blot analysis of exosome core and catalytic subunits enrichments in protrusion and cell body fractions of MDA-MB231 cells. Equal amounts of isolated protrusion and cell body fractions were resolved by SDS-PAGE and blotted for EXOSC5, RRP6, DIS3, as well as VASP (protrusion marker), H2AX (cell body marker) and GAPDH (loading control). While EXOSC5 shows a strong enrichment in protrusion fraction, RRP6 and DIS3 show enrichment in the cell body. (C) Immunofluorescence analysis of exosome core and catalytic subunits localizations. MDA-MB231 mKate-CAAX cells seeded on top of collagen-I coated coverslips were fixed and stained with indicated antibodies. A fraction of EXOSC5 localizes to the leading edge of the cells, while RRP6 and DIS3 are mostly nuclear. (D) RNAi-mediated depletion of exosome core subunits inhibits protrusions. MDA-MB231 mKate CAAX cells were transfected with indicated siRNAs. After 72 hr, the cells were seeded on top of transwell filters for 4 hr before being fixed and analyzed by confocal microscopy. Images show protrusions at the bottom of the filter. Red, cell membranes; blue, filter. Scale bar, 50 μm. See also Movie S6. (E) Quantification of protrusions from (D) (n = 10). Significant p values are stated above each bar graph. Error bars are SD. (F) Exosome core depletion switches MDA-MB231 cell morphology from protrusive to round. MDA-MB231 cells were transfected with indicated siRNAs before being seeded on top of a thick 3D collagen-I matrix 72 hr post-transfection. Cells were imaged 24 hr after seeding. Scale bar, 50 μm. See also Movie S7. (G) Quantification of the percentage of protrusive versus round cells from F (n = 3). Significant p values are stated above each bar graph. Error bars are SD. (H) Inhibition of 3D invasion by knockdown of four different exosome core subunits. MDA-MB231 cells were transfected with indicated siRNAs. After 72 hr, the cells were allowed to invade through a collagen matrix. Cells were labeled 24 hr prior to invasion analysis by CellTracker-CMRA (orange). Z stack images were taken at 5-μm intervals and put together serially from 0 μm to 105 μm. Scale bar, 200 μm. (I) Quantification of 3D invasion analysis from (H) (n = 6). Significant p values are stated above each bar graph. Error bars are SD. See also Figure S3.

To further expand on our results, we validated the role of the exosome core in protrusions, as a potential regulator of local translation and protrusion formation. The exosome core consists of nine subunits that assemble into a two-layered barrel-like structure, with the upper layer forming a cap of three subunits that sit on a ring of six RNase PH domain-containing subunits (Liu et al., 2006). In mammalian cells, the exosome core is catalytically inactive but peripherally associates with a set of catalytically active 3′-5′ exoribonuclease subunits that mediate RNA processing/degradation events (Lykke-Andersen et al., 2009). Although only six subunits of the exosome core were initially included in our screen, we found all of the nine exosome core subunits to be present and more abundant in protrusions than the cell body, with eight out of nine showing at least 2-fold enrichment (Figure 6A). Surprisingly, RRP6 and DIS3, the two catalytic subunits of the exosome that were expressed in our cells, were significantly depleted in protrusions (Figure 6A), suggesting that the activity of the exosome core in protrusions might be independent of the catalytic subunits.

First, we confirmed the enrichment of exosome core in protrusions of MDA-MB231 cells by western blotting. In agreement with the proteomics data, western blot analysis of protrusion and cell body fractions with antibodies against exosome core confirmed a high level of enrichment in protrusions, while blotting for the catalytic subunits showed a strong enrichment in the cell body (Figure 6B). Specificity of the antibodies was validated by RNAi (Figure S3A). Accordingly, immunofluorescence analysis showed a fraction of exosome core that was localized to the leading edge, while the two catalytic subunits of the exosome were enriched in the nucleus (Figure 6C). Next, using RNAi, we depleted each exosome core subunit and assessed the ability of depleted cells to form protrusions through 3-μm transwell filters. Efficient depletion of different exosome core subunits was evaluated by qPCR (Figure S3B), and mass spectrometry (Figure S3C). No apoptosis or change in cell proliferation could be detected upon depletion of exosome core subunits over 72 hr (Figures S3D and S3E), suggesting that transient depletion of the exosome core does not impact cell viability. In these settings, knockdown of seven out of nine exosome core subunits significantly inhibited protrusions (Figures 6D and 6E). Depletion of exosome core subunits also inhibited protrusions in HT-1080 fibrosarcoma cells (Figures S3F and S3G), without impacting cell viability (data not shown), suggesting that the role of the exosome in protrusions is not cell type-specific. Importantly, analysis of protrusion dynamics by time-lapse microscopy revealed that similar to inhibition of local translation, exosome core depletion did not affect protrusion initiation, but resulted in destabilization of already initiated protrusions (Movie S6). Exosome depletion also destabilized protrusions in 3D collagen-I matrix, resulting in conversion of the cells from a polarized mesenchymal-like morphology to round (Figures 6F and 6G; Movie S7). As expected, the invasion of MDA-MB231 cells through 3D collagen-I matrix was also significantly impaired upon depletion of several exosome core subunits (Figures 6H and 6I). Together, these results reveal that the exosome core subunits, but not the catalytic subunits, are enriched in protrusions and are necessary for protrusion stabilization, mesenchymal-like morphology, and migration in 3D.

## Discussion

**Figure 7:**
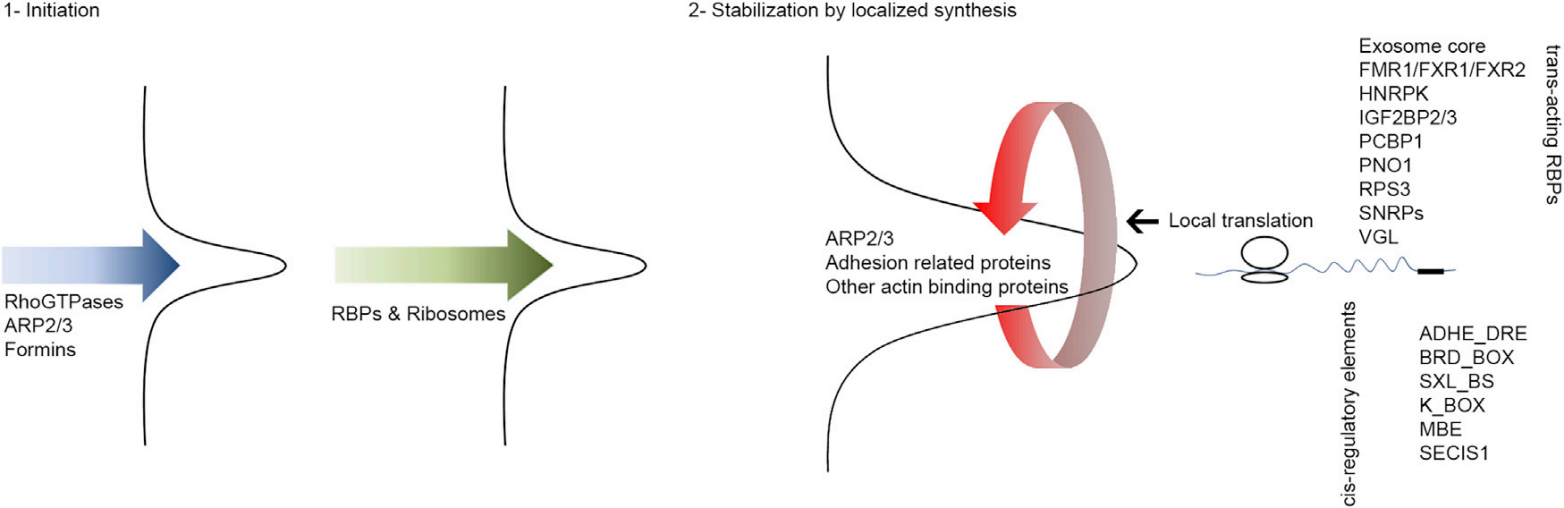
Two-Step Model for Regulation of Protrusion Formation by Local Translation Protrusions are initiated by the action of actin regulators such as Rho-GTPases, which promote actin polymerization via ARP2/3 complex and formins. Once initiated, ribosomes and RBPs in complex with their target mRNAs are transported to protrusions where they mediate local synthesis of more actin-associated proteins, resulting in protrusion stabilization and growth. Local translation rates in protrusions are defined, not by mRNA enrichment alone, but via specific mRNA c*is-*regulatory UTR elements and *trans-*acting RBPs enriched in protrusions.

By comparing the differences between protrusions and the cell body in distribution of proteins, mRNAs, and their relative translation rates, we show that localized translation contributes significantly toward protein localization to protrusions (Figures 2G and 2I). In fact, for the majority of actin-related protein categories enriched in protrusions, such as drivers of actin polymerization, adhesion regulators, and most actin-binding proteins, localized translation seems to be a crucial determinant of protein localization to protrusions (Figure 2H; Data S1E). However, unlike actin-related proteins, RBPs and ribosomal proteins exhibit much higher relative protein levels in protrusions compared to their relative local translation rates (Figure 2H; Data S1E), suggesting that their localization to protrusions must be independent of local synthesis and thus defined by transport. In case of the majority of ribosomal proteins, this is not surprising as regardless of where they may be synthesized in the cytoplasm, they would have to be first transported to the nucleus where they associate with rRNAs in order to form the ribosome subunits (Kressler et al., 2010). Mature ribosome subunits then need to be transported to other cellular destinations to carry out translation. By analogy, the fact that RBPs are not locally translated either suggests that they may be pre-associated with their target mRNAs before transport to protrusions. Considering this, and as we show that the role of localized translation is to stabilize already initiated protrusions (Figures 3B–3H; Movies S3, S4, and S5), we propose a two-step model for protrusion formation in which ribosomes and specific RBPs in complex with their associated mRNAs are transported to sites of protrusions following protrusion initiation, where their localized activity increases translation of actin cytoskeletal proteins, resulting in further growth and stabilization of already initiated protrusions (Figure 7).

Importantly, we show that mRNA distributions do not correlate with protein distributions between protrusions and the cell body (Figure 2D). In fact, several categories of locally translated proteins in protrusions do not seem to exhibit any mRNA enrichment (Figure 4B). Conversely, several categories of protrusion-enriched mRNAs have low relative translation rates in protrusions, suggestive of their local repression (Figure 4B). These include mitochondrial as well as RNA-binding and ribosomal protein categories, which show a strong enrichment of their mRNAs in protrusions, but these localized mRNAs seem to be translationally repressed (Figure 4B). Regulated translational repression of localized mRNAs has been shown before (Buxbaum et al., 2014, Hüttelmaier et al., 2005). For instance, zipcode binding protein IGF2BP1, which is crucial for targeting β-actin mRNA, also represses translation, but this translational repression can be relieved with the right signal, which in case of IGF2BP1 is phosphorylation at the leading edge by the tyrosine kinase SRC (Hüttelmaier et al., 2005). A similar model may apply to the categories of locally repressed mRNAs defined in this study, which would mean that their local translation could be switched on, following yet unidentified cellular signals. Alternatively, for certain categories of mRNAs, localization to protrusion could be functioning as a means of global translation suppression by keeping them away from their correct site of translational activity in the cell body.

Nevertheless, the lack of a positive correlation between mRNA localization and local translation (Figures 4A and 4B) suggests that the level of mRNA accumulation may not be a good predictor for local translation. A few copies of certain localized mRNAs may be able to support robust levels of local translation, while high levels of localization for other mRNAs may not lead to significant local translation. Although it is possible that our findings may mostly apply to immortalized, transformed, or malignant cells such as the one that was used here, these findings still have important ramifications for RNA localization studies as they highlight that mRNA localization alone may not be an indicator for local protein expression by default.

As mRNA translation is chiefly regulated by *cis*-regulatory elements within 5′ and 3′ UTRs of mRNAs, we investigated the association of different known UTR elements with regulation of local translation in protrusions. Our study reveals that presence of specific 3′ UTR elements is associated with higher local translation rates in protrusions (Figure 4C). The exact functions of these regulatory motifs in control of local translation in protrusions remain to be determined. It also remains to be determined if there are yet unknown UTR elements associated with local translation in protrusions, in addition to the known UTR elements investigated here.

To reveal functionally important RBP regulators of localized translation in protrusion, we performed an RNAi screen, depleting members of RBP categories significantly enriched in protrusions and assessing whether their depletion modulated protrusions. Out of 38 RBPs screened, we identified 17 belonging to different categories that significantly regulated protrusions. Sixteen out of 17 also significantly affected migration in 3D collagen-I matrix (Figure 5D). Many of these RBPs have been reported to regulate RNA localization or local translation. However, a number of proteins we identified, including exosome core subunits, have not been implicated in regulation of RNA targeting or local translation. Previously, the exosome core was shown to be present in both the nucleus and cytosol of eukaryotic cells (Lykke-Andersen et al., 2009), although tagging approaches in *Drosophila* had also suggested presence of certain exosome subunits in the periphery of the cells (Graham et al., 2006). Our data reveal that, at least in a mesenchymal-like highly invasive cancer cell-line, the exosome core, but not its catalytic subunits, is enriched in protrusions (Figures 6A–6C) and acts to stabilize protrusions and promote 3D invasion (Figures 6D–6I; Movies S6 and S7). As cell migration and invasion are believed to be intimately linked with metastasis, the exosome-mediated regulation of mesenchymal-like cell migration described here may impact cancer metastasis. In support of this view, we found that, in human breast cancers, increased expression of the exosome core subunits correlates with bad prognosis. This was the case with every exosome core subunit for which expression data were available (Curtis et al., 2012) (Figures S3H–S3L). This result implicates the exosome core in cancer progression and raises the possibility that regulation of cell migration via the exosome could be resulting in a more aggressive, metastatic phenotype.

It is now evident that many types of non-coding RNAs such as microRNAs, long non-coding RNAs, and anti-sense RNAs play a significant role in post-transcriptional regulation of mRNA expression. It is therefore likely that in addition to RNA-binding proteins, non-coding RNAs may also be involved in regulation of local translation in protrusions. In fact, two of the known *cis*-regulatory elements (K-BOX, BRD-BOX) identified here as associated with higher protrusion translation rates, are not known to bind to any RBPs but instead seem to associate with microRNAs (Lai et al., 2005). As our RNA-seq analysis was limited to mRNAs, it remains to be determined whether certain non-coding RNAs also show enrichment in protrusions, and more importantly, whether they play a functional role in regulating protrusions.

## Experimental Procedures

### Transwell Protrusion, 3D Collagen Invasion, and 3D Collagen Morphology Assays

3D collagen invasion assay and 3D collagen morphology assays were done as described before (Sanz-Moreno et al., 2008) with modifications (see also Supplemental Experimental Procedures). Transwell protrusion formation assays were performed on 3-μm pore transwell filters (6.5 mm insert size, 24-well format), coated with 5 μg/ml collagen. A total of 100,000 cells were seeded in 100 μl serum-free DMEM on top of the filter and 600 μl of DMEM with 10% serum was placed in the bottom chamber. Cells were imaged live, or fixed with 4% formaldehyde at indicated time points before imaging. Multiple tile scans, from at least three independent experiments, each composed of 3 × 3 contiguous fields of view, were taken at two z planes (top and bottom of the filter). For quantification of protrusions, fluorescence intensity at the bottom and top of the filter was then measured by ImageJ and bottom to top ratio was calculated.

### Translation Inhibition and Puromycinylation

For puromycinylation of nascent proteins, cells were treated with 10 μg/ml puromycin for 10 min prior to lysis. For cycloheximide treatments, 10 μg/ml cycloheximide was added to the cells for indicated times. For emetine treatments, indicated dose of emetine was added to the cells for indicated times, followed by a single wash in 10% FBS containing DMEM. For specific inhibition of translation in protrusions, cells were seeded on transwells and allowed to form protrusions for 2 hr. The transwells were transferred to a reservoir dish with 1 μg/ml emetine (or vehicle for control treatment) in 10% FBS containing DMEM. The level of liquid in the reservoir was always significantly lower than on top of the filter, creating a positive hydrostatic pressure that limited emetine diffusion upward. After 5 min, the filters were taken out, washed by dipping the filter in 10% FBS containing DMEM, and transferred back to the original well for downstream analysis.

### Protrusion Purification

Purification of cell protrusions was performed using 3-μm pore polycarbonate transwell filters (75 mm membrane inserts). Filters were coated with 5 μg/ml collagen before being seeded with 10 million cells in serum-free DMEM on top, with DMEM containing 10% serum added to the bottom. For western blot analysis, a single transwell was used per condition. For proteomics analysis, four transwells were used per condition. After allowing the cells to form protrusions for 2 hr, transwells were washed in PBS, fixed with −20°C methanol for 20 min, washed again with PBS, and the protrusions from the bottom of the filter were shaved off using a glass coverslip, with the coverslip being dipped in 2% SDS sample buffer. The cell body fraction was then prepared by direct addition of sample buffer to the top of the filter. A similar procedure was used for RNA purification from protrusions, but from one transwell per condition, no methanol fixing, and lysing in RLT buffer from QIAGEN’s RNeasy Mini Kit. RNA was purified according to the manufacturer’s instructions, followed by spectrophotometric measurement of the quality and concentrations.

### Proteomics/Transcriptomics Analysis

For SILAC labeling, cells were grown for seven doublings in heavy or light SILAC DMEM supplemented with 10% dialyzed FBS and 600 mg/l L-Pro. For pSILAC, light-labeled cells were transiently switched to medium or heavy SILAC DMEM during a 2-hr transwell protrusion formation assay. Reciprocally mixed SILAC or pSILAC samples were resolved by SDS-PAGE, stained with Gel-code blue Coomassie dye (Pierce), and cut into 23 sections before Trypsin digestion and peptide extraction. Liquid chromatography-tandem mass spectrometry (LC-MS/MS) analysis was performed by the Institute of Cancer Research (ICR) proteomics core facility. Mass spectrometry search and quantifications were done by Maxquant software (Cox and Mann, 2008, Cox et al., 2011). For pSILAC quantifications, the L label was ignored and H/M ratios, which measure relative nascent protein levels, were used. For RNA-seq, protrusions and the cell body total RNA preps were generated in duplicates from two biological filter-fractionated replicate experiments. Sample preparations and Illumina sequencings were performed by ICR’s tumor profiling unit. All proteomics/transcriptomics data analyses were performed by Perseus software (Cox and Mann, 2012) (see also Supplemental Experimental Procedures). Mass spectrometry proteomics raw data and search results were deposited to the ProteomeXchange Consortium (Vizcaíno et al., 2014), via the PRIDE partner repository, with the dataset identifiers PRIDE: PXD000914 and PRIDE: PXD002649. RNA-seq data were deposited to the ArrayExpress database (http://www.ebi.ac.uk/arrayexpress) under the accession number ArrayExpress: E-MTAB-2546.

## Author Contributions

F.K.M. and C.J.M. designed all experiments and wrote the manuscript. A.P. assisted with mass spectrometry sample preps and LC-MS/MS runs. H.P. generated the MDA-MB231 mKate CAAX cell line and assisted with confocal microscopy. A.S. assisted with immunofluorescence. S.K. assisted with 3D live-cell imaging. A.M. assisted with exosome validation experiments. Y.Y. performed the gene expression-based stratification of the breast cancer patient survival data. All other experiments and data analyses were performed by F.K.M.

## Accession Numbers

The accession numbers for the mass spectrometry proteomics raw data and search results reported in this paper are PRIDE: PXD000914 and PXD002649. The accession number for the RNA-seq data reported in this paper is ArrayExpress: E-MTAB-2546.

## Supplemental Information

Document S1. Supplemental Experimental Procedures and Figures S1–S3

Movie S1. Protrusions Initiate and Grow Stably through 3-μm Transwell Filter Pores, Related to Figure 1 MDA-MB231 mKate CAAX cells were seeded on collagen-coated 3-m transwell filters and time-lapsed for 5 hr at 5-min intervals as they formed protrusions through the pores. Red, cell membranes; blue, filter. Left image shows protrusions at the bottom of the filter. Right image shows cell bodies at the top of the filter. Scale bar, 10 μm.

Movie S2. Protrusions Initiate and Grow Stably through Pores of 3D Collagen-I Matrix, Related to Figure 1 MDA-MB231 cells were seeded on 3D pepsinized collagen-I gels and time-lapsed for 5–10 hr at 3-min intervals as they formed protrusions. Arrow marks a protrusion that remains stable through the course of imaging. Scale bar, 10 μm.

Movie S3. Protrusions Initiate but Are Not Stable and Retract Back in Cycloheximide-Treated Cells, Related to Figure 3 MDA-MB231 mKate CAAX cells were seeded on collagen-coated 3-μm transwell filters and time-lapsed for 4 hr at 15-min intervals as they formed protrusions through the pores of transwell filters in the presence (right) or absence (left) of 10 μg/ml CHX. Red, cell membranes; blue, filter. Scale bar, 20 μm. The images show protrusions at the bottom of the filter.

Movie S4. Protrusions Initiate but Are Not Stable and Retract Back in Cycloheximide-Treated Cells in 3D Collagen, Related to Figure 3 MDA-MB231 cells were seeded on 3D pepsinized collagen-I gels and time-lapsed for 10 hr at 3-min intervals as they formed protrusions in the presence (right) or absence (left) of 10 μg/ml CHX. Scale bar, 10 μm.

Movie S5. Protrusions Are Destabilized upon Inhibition of Local Translation by Local Emetine Treatment, Related to Figure 3 MDA-MB231 mKate CAAX cells were seeded on collagen-coated 3-μm transwell filters for 2 hr before being treated with 1 μg/ml emetine (right) or mock-treated (left) for 5 min as in Figure S2F. After the treatment, the cells were time-lapsed for 2 hr at 5-min intervals to capture protrusion dynamics. Red, cell membranes; blue, filter. Scale bar, 20 μm. The images show protrusions at the bottom of the filter.

Movie S6. Protrusions Initiate but Are Not Stable and Retract Back in Exosome Core-Depleted MDA-MB231 Cells, Related to Figure 6 Control (left) or EXOSC5-depleted (right) MDAMB231 mKate CAAX cells were seeded on collagen-coated 3-μm transwell filters and time-lapsed for 4 hr at 30-min intervals as they formed protrusions through the pores of transwell filters. Red, cell membranes; blue, filter. Scale bar, 20 μm. The images show protrusions at the bottom of the filter.

Movie S7. Protrusions Initiate but Are Not Stable and Retract Back in Exosome Core-Depleted MDA-MB231 Cells in 3D Collagen, Related to Figure 6 Control (left) or EXOSC5-depleted (right) MDA-MB231 cells were seeded on 3D pepsinized collagen-I gels and time-lapsed for 10 hr at 3-min intervals as they formed protrusions. Scale bar, 10 μm.

Data S1. Proteomics, Transcriptomics, and Local Translation Rate Analysis Datasets, Related to Figure 2 (A) SILAC protein ratios between protrusion and cell body fractions from two reciprocally labelled experiments. (B) Perseus output for 1D annotation enrichment analysis of protein distributions from (A) (Benjamini-Hochberg false detection rate [FDR] = 0.02). Blue, actin cytoskeleton-related categories; green, RNA binding/ribosomal protein categories. (C) RNA-seq fragment per kilobase of transcript per million (FPKM) ratios between protrusion and cell body fractions from two replicate experiments. (D) Pulsed SILAC (H/M) ratios between protrusion and cell body fractions from two reciprocally pulse-labelled experiments. (E) Perseus output for 2D annotation enrichment analysis of protein versus translation rate distributions between protrusions and the cell body (Benjamini-Hochberg FDR = 0.02). Blue, actin cytoskeleton-related categories; green, RNA binding/ribosomal protein categories; red, all organelle-related protein categories.

Data S2. Category Enrichment and UTR Element Analyses, Related to Figure 4 (A) Perseus output for 2D annotation enrichment analysis of mRNA versus translation rate distributions between protrusions and the cell body (Benjamini-Hochberg FDR = 0.02). Blue, actin cytoskeleton-related categories; green, RNA binding/ribosomal protein categories; purple, mitochondrial-related protein categories; red, all other organelle-related protein categories. (B) List of all UTR elements investigated in Figure 4C. Source: UTRscan (Grillo et al., 2010).

Document S2. Article plus Supplemental Information
