# Supplementary material for: Global Analysis of mRNA, Translation, and Protein Localization: Local Translation Is a Key Regulator of Cell Protrusions
Source: Dev Cell. 2015 Nov 9;35(3):344–57. doi: 10.1016/j.devcel.2015.10.005 (PMC4643311; doi:10.1016/j.devcel.2015.10.005)
Supplement: Document S1. Supplemental Experimental Procedures and Figures S1–S3 [file mmc1.pdf]

Developmental Cell
Supplemental Information

Global Analysis of mRNA, Translation, and Protein Localization: Local Translation Is a Key Regulator of Cell Protrusions

Faraz K. Mardakheh, Angela Paul, Sandra Kümper, Amine Sadok, Hugh Paterson, Afshan Mccarthy, Yinyin Yuan, and Christopher J. Marshall

Supplementary Figures and Legends:

Fig. S1. RNA-seq analysis of intracellular mRNA distributions between protrusions and the cell-body, related to Figure 2. A: Schematic representation of RNA-seq based protrusion transcriptomics analysis. **B:** Protrusion and Cell-body localized mRNAs defined by RNA-seq overlap significantly with a previously published microarray based protrusion/cell-body mRNA localization study (Mili et al., 2008). Log2 of Protrusion/Cell-body FPKM ratios from two RNA-seq replicate experiments were plotted against each other. Protrusion (Green), and cell-body (red) localized mRNAs from Mili et al., are highlighted on the graph. P-values for protrusion and cell-body enrichments of Mili et al., mRNAs groups are shown on the graph.

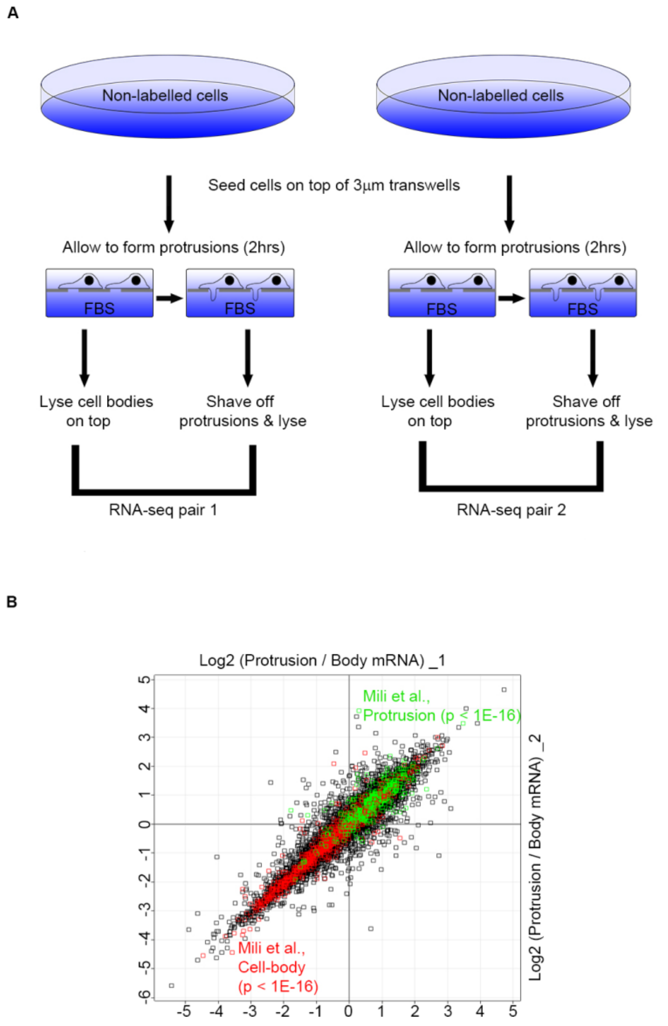

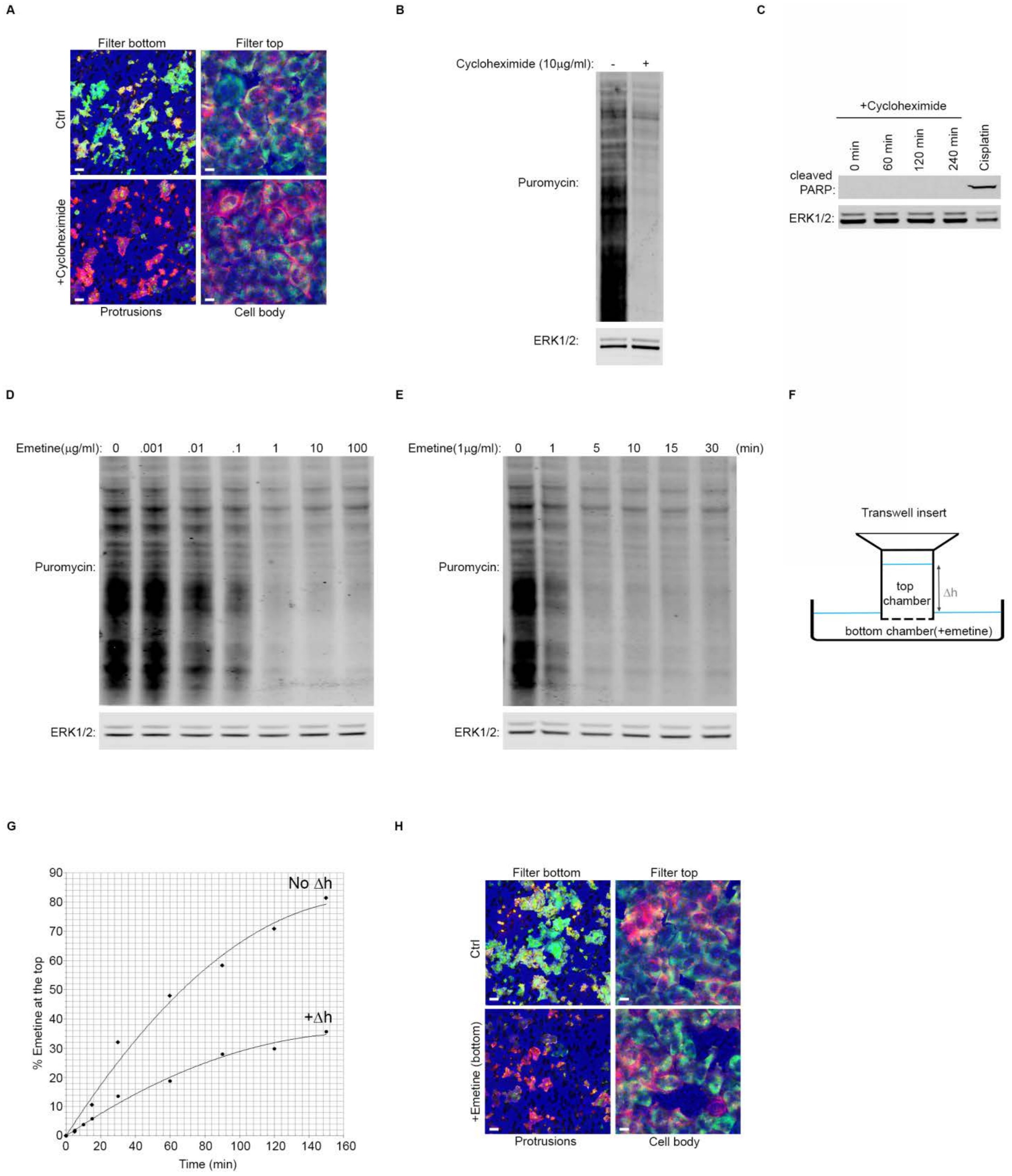

Fig. S2. Analysis of translation inhibition in MDA-MB231 cells, related to Figure 3. A: Protrusions are highly translationally active. MDA-MB231 mKate-CAAX (red) cells were seeded on collagen coated 3μm transwell filters and after 2hrs treated with or without 10μg/ml of

cycloheximide for 10 minutes, followed by puromycin treatment (10μg/ml) for 10 min to label nascent proteins. Afterwards, cells were fixed and visualized by immunofluorescence staining with an anti-puromycin antibody (green). Confocal images were taken from the top (right image) and bottom (left image) of the filter. The filter was visualized by transmitted light (blue). Scale bar = 10μm. Protrusions and the cell bodies show a strong puromycin staining which is abrogated upon cycloheximide treatment. **B:** Cycloheximide inhibition of protein translation in MDA-MB231 cells. MDA-MB231 cells were treated with 10μg/ml of cycloheximide for 10 minutes before being treated with puromycin (10μg/ml) for 10 min to label nascent proteins. Cells were then lysed and resolved by SDS-PAGE and blotted for puromycinylation, and ERK as loading control. **C:** Short-term treatment of MDA-MB231 cells with cycloheximide does not induce apoptosis. MDA-MB231 cells were treated with 10μg/ml of cycloheximide for indicated times before being lysed and analyzed by western blotting with a cleaved-PARP antibody as PARP cleavage marks apoptosis. Total ERK was used as loading control. Cisplatin treatment (20μg/ml for 24 hrs) was used as a positive control for apoptosis induction. **D:** Emetine dose response assessment of translation inhibition. MDA-MB231 cells were treated with indicated doses of emetine for 10 minutes before being washed and treated with puromycin (10μg/ml) to label nascent proteins. Cells were then lysed and resolved by SDS-PAGE and blotted for puromycinylation, and ERK as loading control. Emetine concentrations as low as 1μg/ml significantly inhibit protein translation. **E:** Emetine time-course of translation inhibition. MDA-MB231 cells were treated with 1μg/ml of emetine for indicated times before being washed and treated with puromycin (10μg/ml) to label nascent proteins. Cells were then lysed and resolved by SDS-PAGE and blotted for puromycinylation, and ERK as loading control. Emetine treatment times as short as 5 minutes significantly inhibit protein translation. **F:** Schematic representation

of emetine treatment setting for minimalizing bottom to top emetine diffusion. The level of liquid on top of the filter was kept significantly higher than that of the bottom where emetine was present, in order to create a positive hydrostatic pressure due to height difference (Δh). This slowly pushes the liquids from top to bottom through the transwell pores, thus slowing any upward diffusion from bottom to the top. **G:** Measurement of the diffusion of emetine from the bottom chamber to the top. Concentration of emetine at the top and the bottom of the filter was measured by reading its uv absorbance at 280nm. The amount of emetine on the top was then normalized to the initial amount measured from the bottom. Emetine exhibits a very slow diffusion from bottom to the top under the experimental settings of F, where a height difference (Δh) between the liquid levels is applied. **H:** Translation in protrusions can be specifically inhibited with a 5 minute Emetine treatment (1μg/ml) in the experimental setting of F, without affecting the cell-body translation. MDA-MB231 mKate-CAAX (red) cells were seeded on collagen coated 3μm transwell filters and after 2hrs, the bottom of the filter was treated with or without 1μg/ml of Emetine for 5 minutes as in F, followed by puromycin treatment (10μg/ml) for 10 min to label nascent proteins. Afterwards, cells were fixed and visualized by immunofluorescence staining with an anti-puromycin antibody (green). Confocal images were taken from the top (right image) and bottom (left image) of the filter. The filter was visualized by transmitted light (blue). Scale bar = 10μm. Puromycin staining is wiped out at the bottom of the filter while it is not significantly affected at the top.

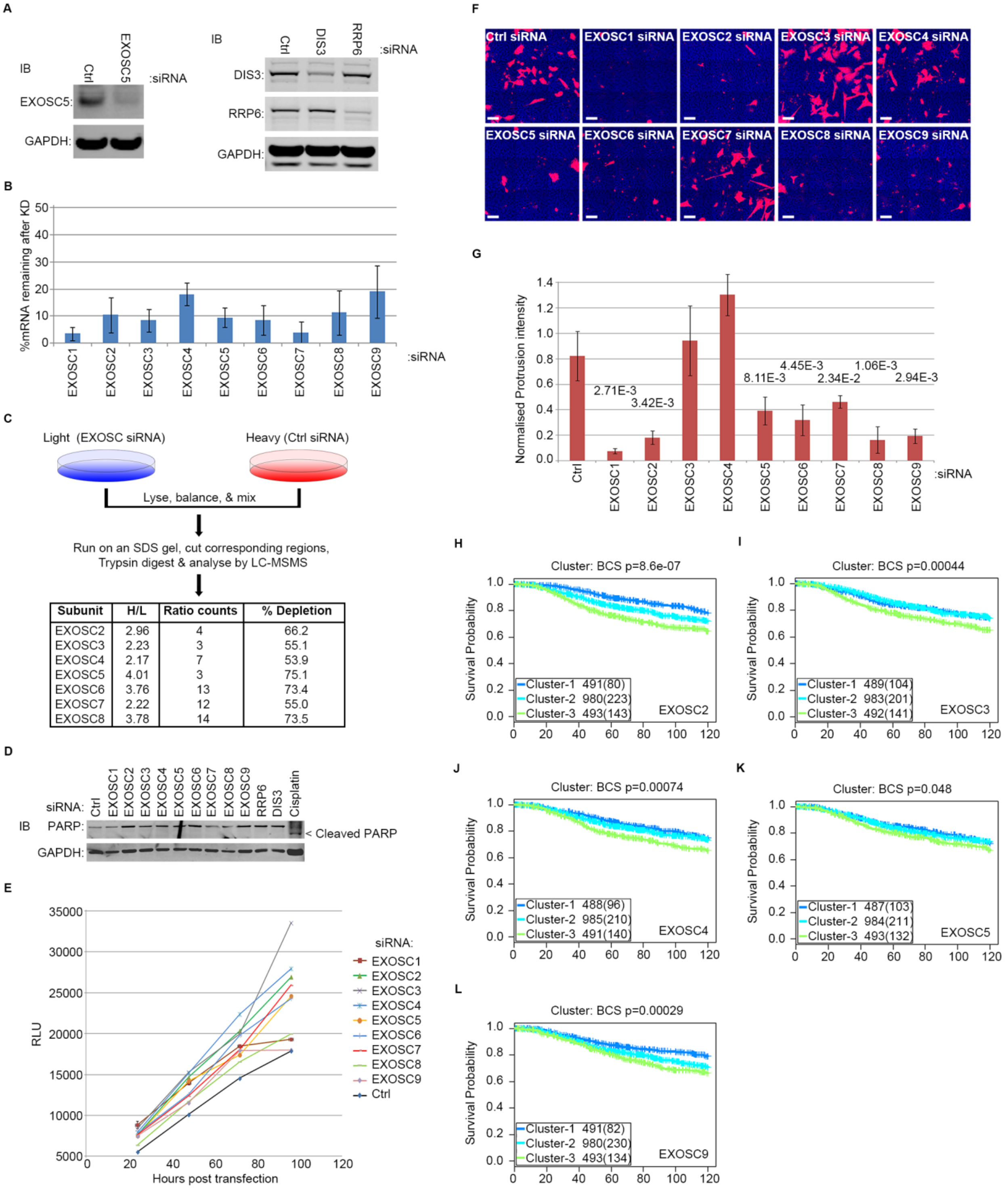

Fig. S3. Analysis of Exosome subunit depletion by siRNA, related to Figure 6. A: Test of exosome core and catalytic subunit antibody specificities. Left: Control and EXOSC5 depleted

MDA-MB231 cell lysates were run and blotted with anti-EXOSC5 antibody. EXOSC5 corresponding band is significantly reduced in EXOSC5 knockdown cells. Right: Control, RRP6, and DIS3 depleted MDA-MB231 cell lysates were run and blotted with anti-RRP6, and anti-DIS3 antibodies. RRP6 and DIS3 corresponding bands are significantly reduced in their respective knockdown cells. GAPDH was used as loading control. **B:** Quantitative PCR verification of siRNA mediated exosome core subunit knockdown at mRNA level in MDA-MB231 cells, 72hrs post-transfection. RNA contents for each exosome subunit knockdown were normalized to GAPDH RNA levels followed by ratio calculation to the non-targeting control values. All error bars are SD. **C:** SILAC based quantitative mass spectrometric verification of siRNA mediated exosome core subunit knockdowns at the protein level in MDA-MB231 cells, 72hrs post-transfection. Knockdown of each exosome core subunit was performed individually and in light SILAC labeled cells. After lysis, these knockdown lysates were then mixed with an equal amount of heavy SILAC labelled control siRNA knockdown lysates. ~10µg of each SILAC mix was then run on an SDS-PAGE gel, and the approximate region corresponding to the exosome subunits molecular weight was cut, trypsin digested, and analyzed by LC-MS/MS. Out of the total of 9 exosome core subunits, 7 were successfully identified and quantified. % of KD was then estimated from each calculated normalized H/L SILAC ratios. **D:** Short-term knockdown of exosome subunits does not induce apoptosis. MDA-MB231 cells were transfected with control vs. indicated siRNA of exosome core as well as RRP6 and DIS3 catalytic subunits. After 72hrs, the cells were lysed and analysed by western blotting for PARP cleavage which marks apoptosis. Cisplatin treatment (20µg/ml for 24 hrs) was used as a positive control. **E:** Short-term knockdown of exosome core subunits does not affect cell proliferation. MDA-MB231 cells were transfected with control siRNAs or siRNA against exosome core subunits. Viability

was then assessed at 24, 48, 72, and 96 hrs post transfection by CellTiter-Glo. All cells show linear and comparable growth rates apart from EXOSC1 and EXOSC9 which show a decrease but only at 96hrs post transfection. RLU: Random Luminescence Unit. **F:** Exosome core depletion inhibits protrusions in HT1080 cells. HT1080 Fibrosarcoma cells were transfected with control or exosome core subunit siRNAs. After 72hrs, the cells were seeded on collagen coated 3µm transwell filters to form protrusions before being fixed 4hrs later and analyzed by confocal microscopy. Cells were stained 24hrs prior to seeding by CellTracker-CMRA (red). Filter was visualized by transmitted light (blue). Scale bar = 50µm. The images show protrusions at the bottom of the filter. **G:** Quantification of protrusions from F (n = 4). All error bars are SD. **H:** Increased expression of exosome core subunit EXOSC2 correlates with bad prognosis in breast cancers. Kaplan-Meier plot of disease-specific survival (truncated at 10 years) for stratification by EXOSC2 gene expression in 1,980 breast cancer patients (Curtis et al., 2012). The dataset was divided into 3 clusters. Cluster-1 had the 25% lowest expression (blue). Cluster-3 had the 25% highest expression (green), and cluster-2 had the expression levels in between (cyan). For each cluster, the number of samples at risk is indicated as well as the total number of deaths (in parentheses). **I:** Increased expression of exosome core subunit EXOSC3 correlates with bad prognosis in breast cancers. Kaplan-Meier plot of disease-specific survival (truncated at 10 years) for stratification by EXOSC3 gene expression in 1,980 breast cancer patients as in H. **J:** Increased expression of exosome core subunit EXOSC4 correlates with bad prognosis in breast cancers. Kaplan-Meier plot of disease-specific survival (truncated at 10 years) for stratification by EXOSC4 gene expression in 1,980 breast cancer patients as in H. **K:** Increased expression of exosome core subunit EXOSC5 correlates with bad prognosis in breast cancers. Kaplan-Meier plot of disease-specific survival (truncated at 10 years) for stratification by EXOSC5 gene

expression in 1,980 breast cancer patients as in H. **L:** Increased expression of exosome core subunit EXOSC9 correlates with bad prognosis in breast cancers. Kaplan-Meier plot of disease-specific survival (truncated at 10 years) for stratification by EXOSC9 gene expression in 1,980 breast cancer patients as in H.

Supplemental Data Files, and Movie Legends:

Supplemental Data File 1: Proteomics, transcriptomics, and local translation rate analysis datasets, related to Figure 2. **A:** SILAC protein ratios between protrusion and cell-body fractions from two reciprocally labelled experiments. **B:** Perseus output for 1D annotation enrichment analysis of protein distributions from 1A (Benjamini-Hochberg FDR = 0.02). Actin cytoskeleton related categories are marked in blue; RNA binding/Ribosomal protein categories are marked in green. **C:** RNA-seq FPKM ratios between protrusion and cell-body fractions from two replicate experiments**.** **D:** Pulsed SILAC (H/M) ratios between protrusion and cell-body fractions from two reciprocally pulse-labelled experiments **E:** Perseus output for 2D annotation enrichment analysis of Protein versus translation rate distributions between protrusions and the cell-body (Benjamini-Hochberg FDR = 0.02). Actin cytoskeleton related categories are marked in blue; RNA binding/Ribosomal protein categories are marked in green; all organelle related protein categories are marked in red.

Supplemental Data File 2: Category enrichment and UTR element analyses, related to Figure 4: A: Perseus output for 2D annotation enrichment analysis of mRNA versus translation rate distributions between protrusions and the cell-body (Benjamini-Hochberg FDR = 0.02). Actin cytoskeleton related categories are marked in blue; RNA binding/Ribosomal protein categories are marked in green; Mitochondrial related protein categories are marked in purple; all other organelle related protein categories are marked in red. **B:** List of all UTR elements investigated in Figure 4C (Source: UTRscan (Grillo et al., 2010)).

Movie S1: (related to Figure 1) Protrusions initiate and grow stably through 3μm transwell filter pores. MDA-MB231 mKate CAAX cells were seeded on collagen coated 3μm transwell filters and time-lapsed for 5hrs at 5 minute intervals as they formed protrusions through the pores. Red: Cell membranes, Blue: filter. Left image shows protrusions at the bottom of the filter. Right image shows cell-bodies at the top of the filter. Scale bar = 10μm

Movie S2: (related to Figure 1) Protrusions initiate and grow stably through pores of 3D collagen-I matrix. MDA-MB231 cells were seeded on 3D pepsinised collagen-I gels and time-lapsed for 5-10 hrs at 3 minute intervals as they formed protrusions. Arrow marks a protrusion that remains stable through the course of imaging. Scale bar = 10μm

Movie S3: (related to Figure 3) Protrusions initiate but are not stable and retract back in Cycloheximide (CHX) treated cells. MDA-MB231 mKate CAAX cells were seeded on collagen coated 3μm transwell filters and time-lapsed for 4hrs at 15 minute intervals as they

formed protrusions through the pores of transwell filters in the presence (right) or absence (left) of 10μg/ml CHX. Red: Cell membranes, Blue: filter. Scale bar = 20μm. The images show protrusions at the bottom of the filter.

Movie S4: (related to Figure 3) Protrusions initiate but are not stable and retract back in Cycloheximide (CHX) treated cells in 3D collagen. MDA-MB231 cells were seeded on 3D pepsinised collagen-I gels and time-lapsed for 10 hrs at 3 minute intervals as they formed protrusions in the presence (right) or absence (left) of 10μg/ml CHX. Scale bar = 10μm.

Movie S5: (related to Figure 3) Protrusions are destabilized upon inhibition of local translation by local emetine treatment. MDA-MB231 mKate CAAX cells were seeded on collagen coated 3μm transwell filters for 2 hrs before being treated with 1μg/ml emetine (right) or mock treated (left) for 5 minutes as in Fig. S2F. After the treatment, the cells were time-lapsed for 2hrs at 5 minute intervals to capture protrusion dynamics. Red: Cell membranes, Blue: filter. Scale bar = 20μm. The images show protrusions at the bottom of the filter.

Movie S6: (related to Figure 6) Protrusions initiate but are not stable and retract back in exosome core depleted MDA-MB231 cells. Control (left) or EXOSC5 depleted (right) MDA-MB231 mKate CAAX cells were seeded on collagen coated 3μm transwell filters and time-lapsed for 4hrs at 30 minute intervals as they formed protrusions through the pores of transwell filters. Red: Cell membranes, Blue: filter. Scale bar = 20μm. The images show protrusions at the bottom of the filter.

Movie S7: (related to Figure 6) Protrusions initiate but are not stable and retract back in exosome core depleted MDA-MB231 cells in 3D collagen. Control (left) or EXOSC5 depleted (right) MDA-MB231 cells were seeded on 3D pepsinised collagen-I gels and time-lapsed for 10 hrs at 3 minute intervals as they formed protrusions. Scale bar = 10μm.

Supplemental Experimental Procedures:

Antibodies, reagents, and plasmids:

Rabbit monoclonal antibodies against ERK1/2 (137F5), VASP (3132), H2AX (7631), PARP (46D11), and cleaved PARP (D64E10), were all from Cell Signaling Technology. Mouse monoclonal anti-GAPDH antibody was from Novus Biologicals. Mouse monoclonal anti-puromycin (12D10) was from Millipore. Rabbit polyclonal anti-EXOSC5 antibody was a gift from Ger Pruijn (Department of Biomolecular Chemistry, Radboud University Nijmegen, Nijmegen, The Netherlands). Mouse monoclonal and rabbit polyclonal antibodies against DIS3 and RRP6 were from Abcam. Hoechst 33258 pentahydrate, CellTracker-CMFDA, CellTracker-CMRA, CellTrace Far Red DDAO-SE, and Alexa Fluor-488 Phalloidin were from Life Technologies Molecular Probes. All secondary antibodies were also from Life Technologies Molecular Probes. Cycloheximide and Emetine dihydrochloride hydrate were from Sigma-Aldrich. All siRNAs (siGENOME Smartpool) were purchased from Dharmacon. Quantitative-PCR probes were purchased from Primer Design and Qiagen. Polycarbonate transwell filters were from Corning. Bovine Collagen (PureCol, 3mg/ml) was from Advanced BioMatrix. CellTiter-Glo was purchased from Promega. Membrane targeted mKate (pmKate2-f-mem) expression construct was purchased from Evrogen.

Cell staining, Immunofluorescence, and confocal microscopy

Staining with CellTracker dyes was performed according to manufacturer's instructions 24 hrs prior to analysis. CellTracker-CMFDA was used at 1.25μM, and CellTracker-CMRA at 5μM. CellTrace Far Red DDAO-SE staining of collagen coated filters was also done 24hrs prior to

analysis. Filters were stained with 100μM dye in PBS for 20 minutes followed by 3 PBS washes and kept in PBS at 4°C in darkness before seeding of the cells. For immunofluorescence staining on coated cover slips or transwells, cells were seeded and fixed after 2hrs (or as indicated otherwise) with PBS / 4%Formaldehyde, permeabilised by PBS / 0.2% Triton, and blocked in PBS / 4%BSA for 20 minutes before being incubated with primary and secondary antibodies (1:200) for one hour in blocking buffer. 3 x PBS washes were performed in between each antibody step. Phalloidin (1:100) and Hoechst (1:5000) staining was also done in blocking buffer for 20 minutes. Time-lapse analysis on protrusions in 3D collagen was performed on a Nikon Eclipse TE2000-S inverted epi-fluorescence microscope with a Hamamatsu cooled CCD camera using a 20x NA objective. Immunofluorescence and transwell imaging were performed on a Ziess LSM 710 confocal microscope with a 63x NA oil objective at optimal aperture settings. 4 times averaging per image was used. For time-lapse confocal analysis, the filter was placed on a glass bottomed 35mm dish containing DMEM with 10% serum right after seeding, and imaged in a live imaging chamber (37°C/10%CO_2) by confocal microscopy for indicated times. A tile scan of 3x3 contiguous fields of view was taken at 2 Z-planes (top and bottom of the filter) as before. In both fixed and live conditions, the filter was either visualized by collagen staining with CellTrace Far Red DDAO-SE, or by confocal transmitted light imaging.

3D collagen invasion, and 3D collagen morphology assays:

For 3D collagen invasion assay cells were suspended in serum-free collagen (2.3mg/ml) to a final concentration of 10,000cells/100μl. For each condition, 100μl aliquots were dispensed into 96-well ViewPlates (Perkin-Elmer) pre-coated with 0.2% fatty acid free BSA. 4 to 8 wells were used per condition. Plates were then centrifuged at 300g to collect the cells at the bottom of the

wells, and incubated at 37°C/10% CO2 for 3hrs to let the collagen set into a gel. 30µl DMEM with 10% serum was then added to the top and cells were left to invade up through the collagen gel for 24hrs before being fixed and stained with Hoechst (5µg/ml). The plates were then imaged by an Operetta High Content Imaging System (PerkinElmer) using Z-planes at 0µm, 30µm, & 60µm. The invasion index was calculated as the number of cells at 30µm and 60 µm divided by the total number of cells. For 3D imaging of invaded cells, cells were stained by CellTracker-CMRA 24hrs prior to invasion assays. Following fixation, sequential Z-sections of the collagen embedded cells were obtained at 5µm intervals from 0µm to 105µm. For morphology analysis on 3D collagen (1.7mg/ml bovine pepsinised in DMEM), cells were seeded in 10% serum containing DMEM and allowed to adhere before treatment and imaging on a phase contrast microscope using the 10x objective (Nikon).

RNAi screening of RNA Binding Domain (RBD) containing proteins

For RNAi screening of RBD containing proteins, 10,000 cells were seeded on top of collagen coated 96-well HTS transwell filters (3µm pore) without media in the bottom chamber, and transfected the next day with the siRNA library (10nM). After 72hrs, media on the cells was replaced with 70µl serum-free DMEM and 200µl DMEM with 10% serum was added to the bottom chamber of each well to start protrusion formation. After 4hrs, cells were fixed and imaged by confocal microscopy. Four 3x3 tile scans from two wells per each siRNA were used.

Mass spectrometry sample preparation and LC-MS/MS analysis

For in-gel digestion, excised gel sections were diced and reduced with 5 mM TCEP, alkylated with 55 mM iodoacetamide, and trypsin digested with modified porcine Trypsin (Promega) at

37°C overnight. After digestion, peptides were extracted with acetonitrile and triethylammonium bicarbonate washes. The solution was dried in a speedvac, and reconstituted in 2% acetonitrile/0.1% formic acid for LC-MS/MS. LC-MS/MS runs were performed by ICR's proteomics core facility. Briefly, 40% of each fraction was analysed as 4μL injections using HP1200 reversed phase chromatography platform (Agilent). Peptides were resolved on a 75 μm I.D. C18 Pepmap column with 3 μm particle size (LC Packings/Dionex) over 90 min using a linear gradient of 96:4 to 50:50 buffer A:B (buffer A: 2% acetonitrile/0.1% formic acid; buffer B: 80% acetonitrile/0.1% formic acid) at 300nL/min. Peptides were ionized by electrospray ionization using 1.9 kV applied directly to the post-column LC eluent via a microtee built into the nanospray source. Sample was infused into an LTQ Velos Orbitrap mass spectrometer (Thermo Fisher Scientific) using a 20 μm I.D., 10 μm tapered tip non-coated SilicaTip emitter (New Objectives). The ion transfer tube was heated to 200°C and the S-lens set to 60%. MS/MS were acquired using data dependent acquisition to sequence the top 20 most intense ions using standard ion trap scans. Automatic gain control was set to 1,000,000 for FT-MS and 30,000 for IT-MS/MS, full FT-MS maximum inject time was 500ms and normalized collision energy was set to 35% with an activation time of 10ms. Wideband activation was used to co-fragment precursor ions undergoing neutral loss of up to -20 m/z from the parent ion, including loss of water/ammonia. MS/MS was acquired for selected precursor ions with a single repeat count followed by dynamic exclusion with a 10ppm mass window for 15s based on a maximal exclusion list of 500 entries.

Proteomics search and SILAC quantifications

All mass spectrometry raw files were searched and quantified by Maxquant software (Cox and Mann, 2008; Cox et al., 2011). The search was performed using the Andromeda search engine, against the Human IPI database (version 3.68). Mass tolerance filters of 6ppm and 0.5Da were used for precursor and fragment masses, respectively. A minimum peptide length of 6 amino acids was used. Second-peptide search, and match between runs (2 minutes window) option, were all enabled. For total SILAC, but not the pulsed-SILAC (pSILAC) analysis, the re-quantify option was also enabled. Methionine oxidation and N-terminal acetylation were added as variable modifications while carbamidomethylation was considered as a fixed modification on Cysteine. A maximum of 2 missed cleavages were allowed, and the false discovery rate (FDR) was set at 0.01 for both peptide and protein identifications. For total SILAC quantifications, an overall minimum ratio count (H/L) of 2 per protein was used. For pSILAC, this was set to 1 (H/M). Only razor or unique unmodified peptides as well as Methionine oxidized and N-terminally acetylated peptides were used for protein quantifications. Data analysis on the search results was performed by Perseus software (Cox and Mann, 2012), using the Maxquant 'protein groups' output file as input. The median subtracted normalized ratios were used for all downstream data analyses.

RNA-seq sample prep, sequencing, and analysis

Sample prep and Illumina sequencing were performed by ICR's tumor profiling unit. Briefly, total RNA quality was assessed using the 2100 Bioanalyzer (Agilent). Poly-A mRNA was then isolated from the total RNA sample using the Dynabeads mRNA Purification Kit (Life Technologies). Purified mRNAs were fragmented using the mRNA Fragmentation Reagents (Life Technologies) and ethanol precipitated. The size range of generated fragments was kept at

60 to 1000 nucleotides, with a peak at around 150 nucleotides. To make cDNA from the fragmented RNA samples, NEBNext mRNA Library Prep Reagent Set for Illumina was used (NEB), with the synthesis being performed Strand-wise (first-strand synthesis then second-strand synthesis) followed by purification using QIAquick Columns (Qiagen). End Repair, dA-tailing, and Adapter ligation were all performed based on NEBNext mRNA Library Prep Reagent Set for Illumina instructions. Agarose gel separation was used to select for the appropriate library size. To get a library size of ≈400bp (insert size ≈330bp) a 350-500bp slice was taken from each of the samples. Gel extraction was then performed using Qiagen MinElute gel extraction kit (Qiagen). To enrich for the correctly adapter-ligated and size-selected cDNA, a PCR was performed using the Herculase II polymerase enzyme (Agilent), also adding the specific indexes for multiplexing. The PCR was then followed by an AMPure bead purification to exclude primer-dimers and other smaller fragments from the final library. The quantity and quality of the prepared library was evaluated on Bioanalyzer and the samples were quantified using qPCR. Subsequently, libraries were combined for multiplexed sequencing runs. For sequencing Read-1 and Read-2, the sequencing primers provided with the PE Cluster Generation kits were used. RNA-seq data was acquired on a HiSeq2000 (Illumina) sequencer, generating 2 x 76 bp reads. Sequences were then processed by ICR's tumor profiling unit (FASTQ file creation, de-multiplexing, filtering failed reads, and Tophat analysis) and a single tab-delimited list of mRNAs with their corresponding Fragment Per Kilo-base per Million (FPKM) values was generated. Hits with FPKM values less than 1 were filtered out. Any non-coding sequences were also filtered out. Protrusion to cell-body FPKM ratios were calculated for each biological replicate. All downstream data analysis was then performed using Perseus (Cox and Mann, 2012).

Graphs, bioinformatics, and statistical analysis

All proteomics and RNA-seq graphs were generated by Perseus (Cox and Mann, 2012). All Bioinformatics analyses of proteomics and RNA-seq datasets were also performed by Perseus. The annotation enrichment analysis was performed using 1D or 2D annotation enrichment algorithms. A Benjamini–Hochberg false discovery rate (FDR) of 0.02 was used to correct for annotation enrichment p-values. For UTR analysis, the data was annotated for different UTR elements using UTRref database and UTRscan tool (Grillo et al., 2010). Pearson correlation coefficients and p-values for correlations were also generated with Perseus. All bar graph were generated by Microsoft Excel. For protrusion, morphology, and migration assays, P-values were calculated against control populations using two-tailed, heteroscedastic t-test analysis. All error bars are standard deviation (SD).

References

Cox, J., and Mann, M. (2008). MaxQuant enables high peptide identification rates, individualized p.p.b.-range mass accuracies and proteome-wide protein quantification. Nat Biotechnol *26*, 1367-1372.

Cox, J., and Mann, M. (2012). 1D and 2D annotation enrichment: a statistical method integrating quantitative proteomics with complementary high-throughput data. BMC Bioinformatics *13 Suppl 16*, S12.

Cox, J., Neuhauser, N., Michalski, A., Scheltema, R.A., Olsen, J.V., and Mann, M. (2011). Andromeda: a peptide search engine integrated into the MaxQuant environment. J Proteome Res *10*, 1794-1805.

Curtis, C., Shah, S.P., Chin, S.F., Turashvili, G., Rueda, O.M., Dunning, M.J., Speed, D., Lynch, A.G., Samarajiwa, S., Yuan, Y.*, et al.* (2012). The genomic and transcriptomic architecture of 2,000 breast tumours reveals novel subgroups. Nature *486*, 346-352.

Grillo, G., Turi, A., Licciulli, F., Mignone, F., Liuni, S., Banfi, S., Gennarino, V.A., Horner, D.S., Pavesi, G., Picardi, E.*, et al.* (2010). UTRdb and UTRsite (RELEASE 2010): a collection of sequences and regulatory motifs of the untranslated regions of eukaryotic mRNAs. Nucleic acids research *38*, D75-80.

Mili, S., Moissoglu, K., and Macara, I.G. (2008). Genome-wide screen reveals APC-associated RNAs enriched in cell protrusions. Nature *453*, 115-119.
