# Supplementary figures and images for: Global Analysis of mRNA, Translation, and Protein Localization: Local Translation Is a Key Regulator of Cell Protrusions
Source: Dev Cell. 2015 Nov 9;35(3):344–57. doi: 10.1016/j.devcel.2015.10.005 (PMC4643311; doi:10.1016/j.devcel.2015.10.005)

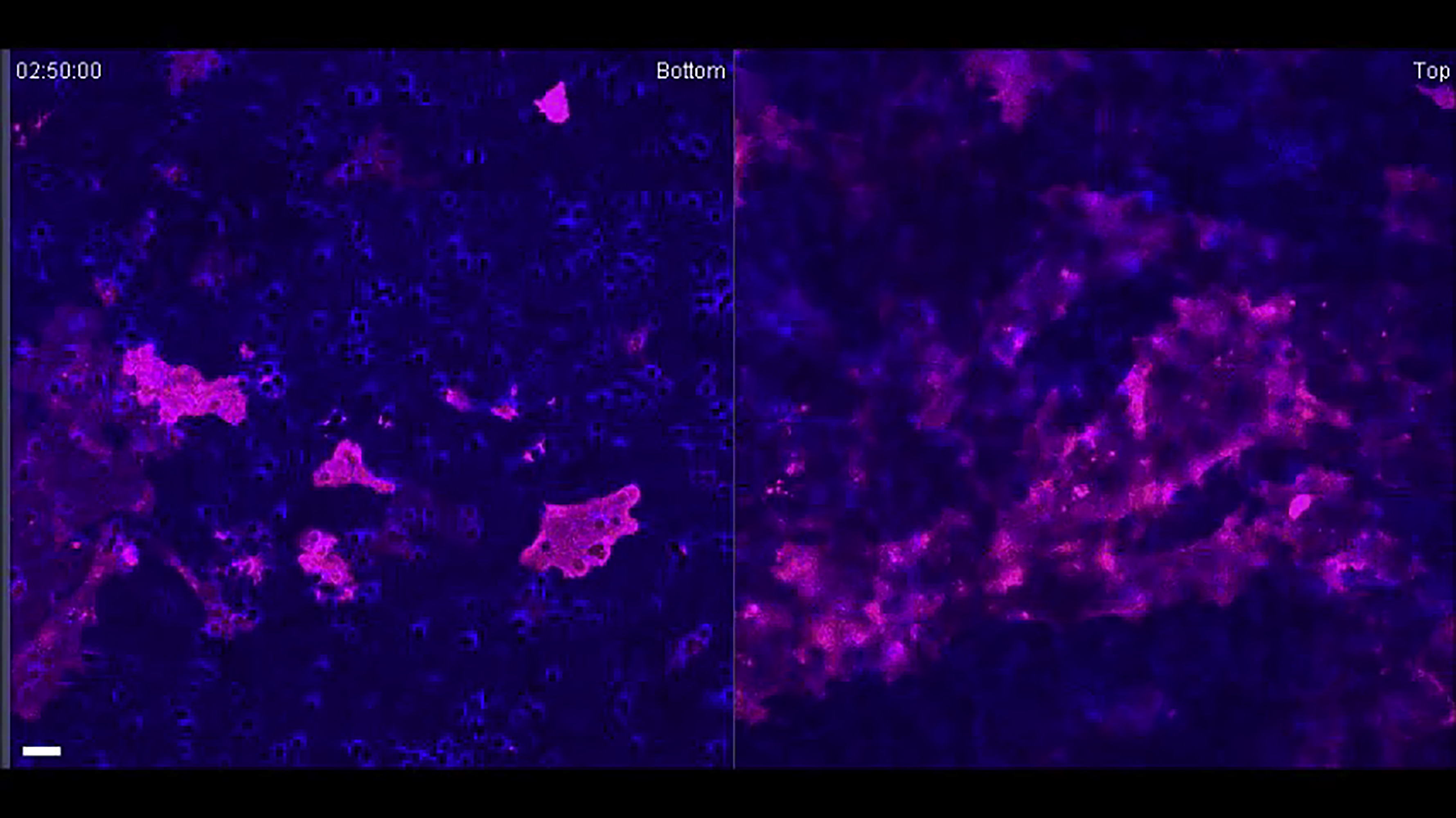

Supplement: Movie S1. Protrusions Initiate and Grow Stably through 3-μm Transwell Filter Pores, Related to Figure 1 — MDA-MB231 mKate CAAX cells were seeded on collagen-coated 3-m transwell filters and time-lapsed for 5 hr at 5-min intervals as they formed protrusions through the pores. Red, cell membranes; blue, filter. Left image shows protrusions at the bottom of the filter. Right image shows cell bodies at the top of the filter. Scale bar, 10 μm. [file mmc2.jpg]

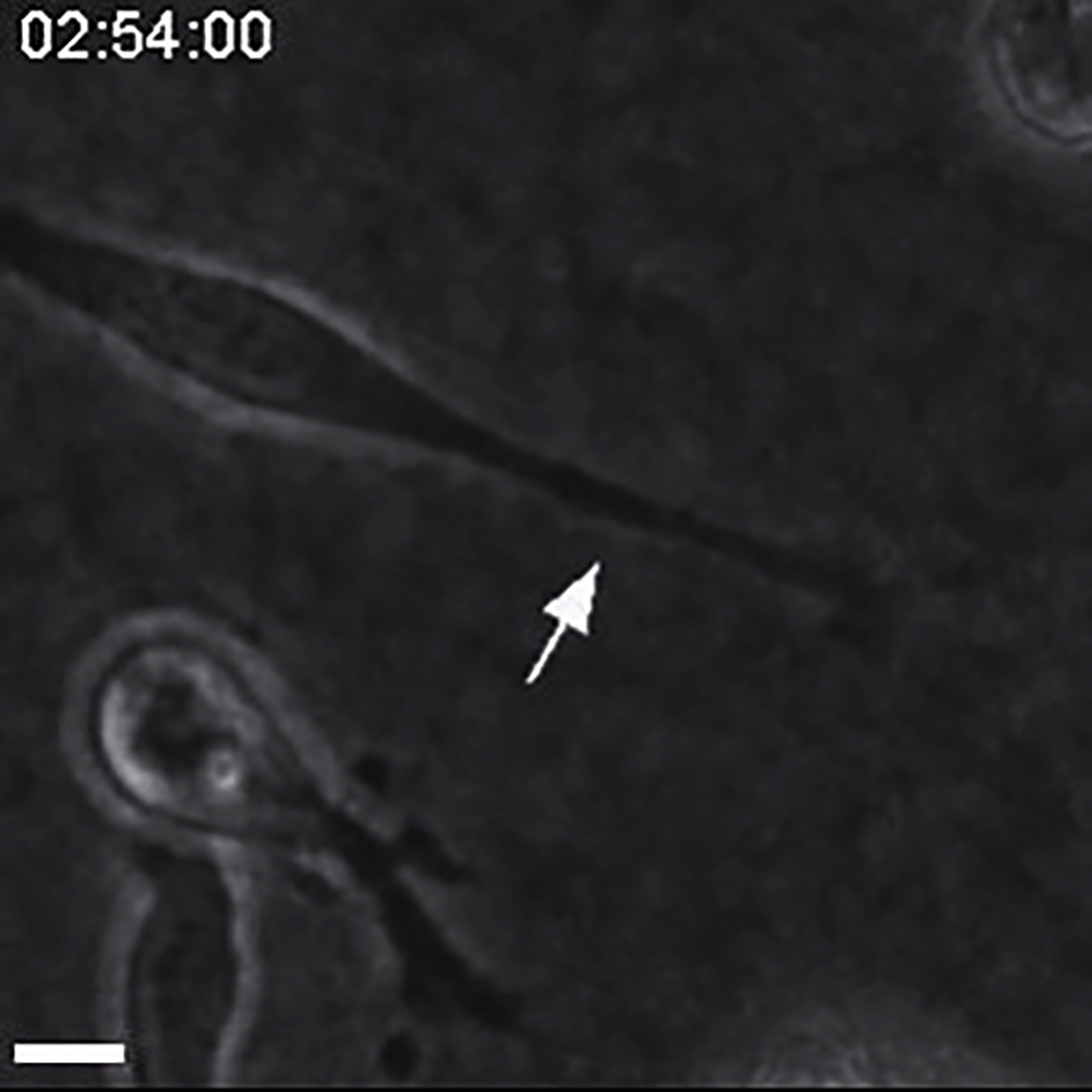

Supplement: Movie S2. Protrusions Initiate and Grow Stably through Pores of 3D Collagen-I Matrix, Related to Figure 1 — MDA-MB231 cells were seeded on 3D pepsinized collagen-I gels and time-lapsed for 5–10 hr at 3-min intervals as they formed protrusions. Arrow marks a protrusion that remains stable through the course of imaging. Scale bar, 10 μm. [file mmc3.jpg]

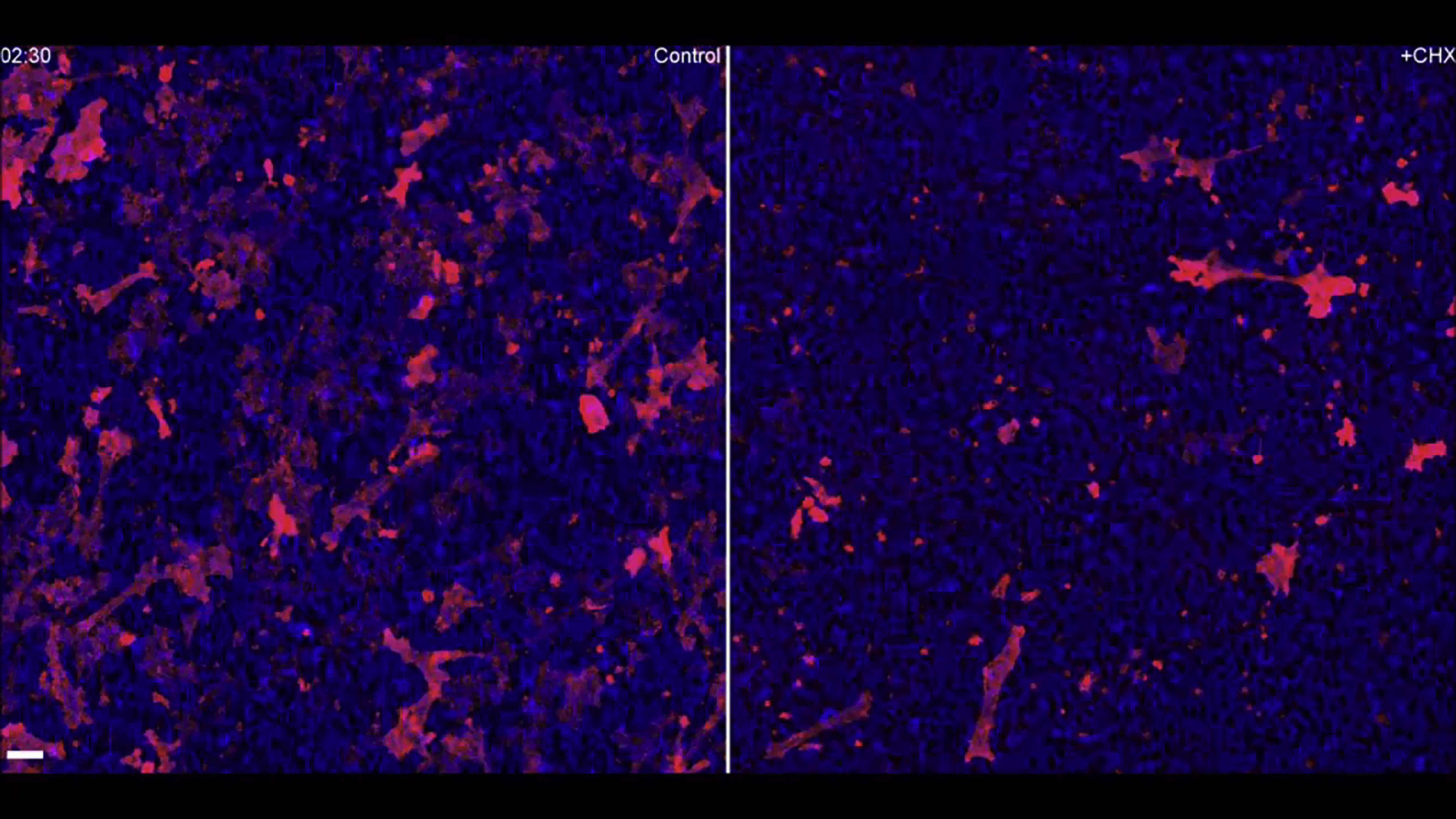

Supplement: Movie S3. Protrusions Initiate but Are Not Stable and Retract Back in Cycloheximide-Treated Cells, Related to Figure 3 — MDA-MB231 mKate CAAX cells were seeded on collagen-coated 3-μm transwell filters and time-lapsed for 4 hr at 15-min intervals as they formed protrusions through the pores of transwell filters in the presence (right) or absence (left) of 10 μg/ml CHX. Red, cell membranes; blue, filter. Scale bar, 20 μm. The images show protrusions at the bottom of the filter. [file mmc4.jpg]

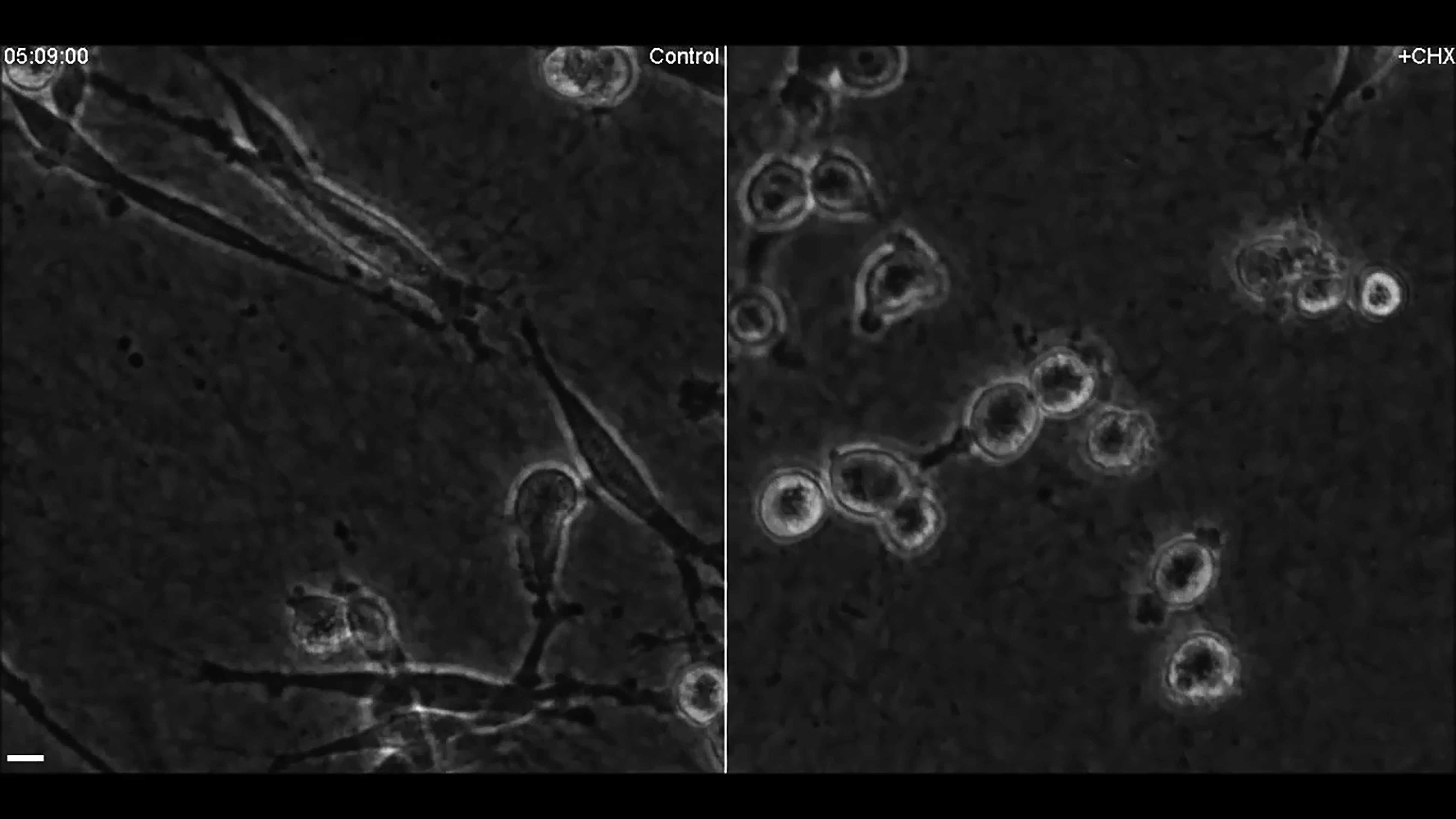

Supplement: Movie S4. Protrusions Initiate but Are Not Stable and Retract Back in Cycloheximide-Treated Cells in 3D Collagen, Related to Figure 3 — MDA-MB231 cells were seeded on 3D pepsinized collagen-I gels and time-lapsed for 10 hr at 3-min intervals as they formed protrusions in the presence (right) or absence (left) of 10 μg/ml CHX. Scale bar, 10 μm. [file mmc5.jpg]

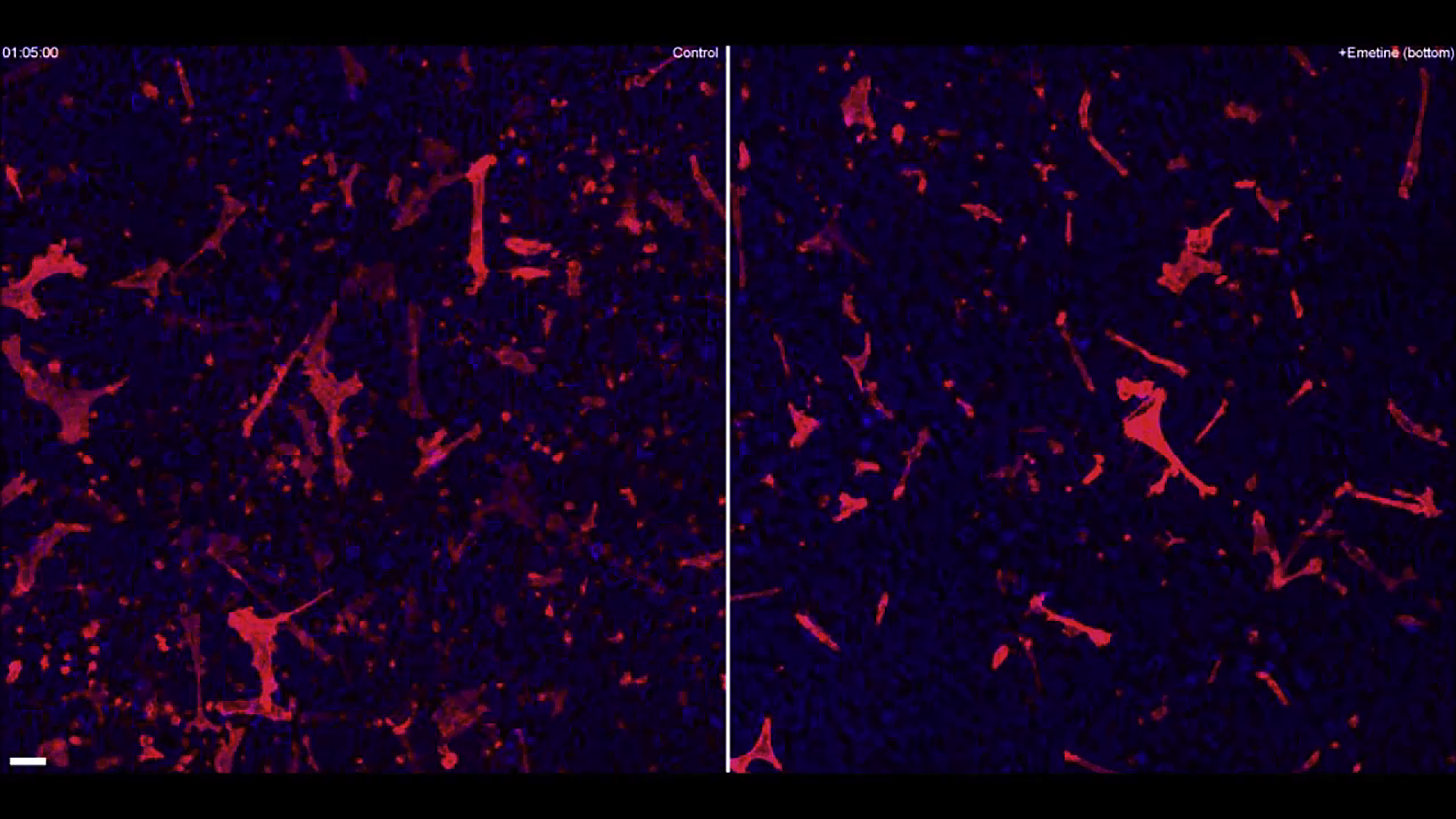

Supplement: Movie S5. Protrusions Are Destabilized upon Inhibition of Local Translation by Local Emetine Treatment, Related to Figure 3 — MDA-MB231 mKate CAAX cells were seeded on collagen-coated 3-μm transwell filters for 2 hr before being treated with 1 μg/ml emetine (right) or mock-treated (left) for 5 min as in Figure S2F. After the treatment, the cells were time-lapsed for 2 hr at 5-min intervals to capture protrusion dynamics. Red, cell membranes; blue, filter. Scale bar, 20 μm. The images show protrusions at the bottom of the filter. [file mmc6.jpg]

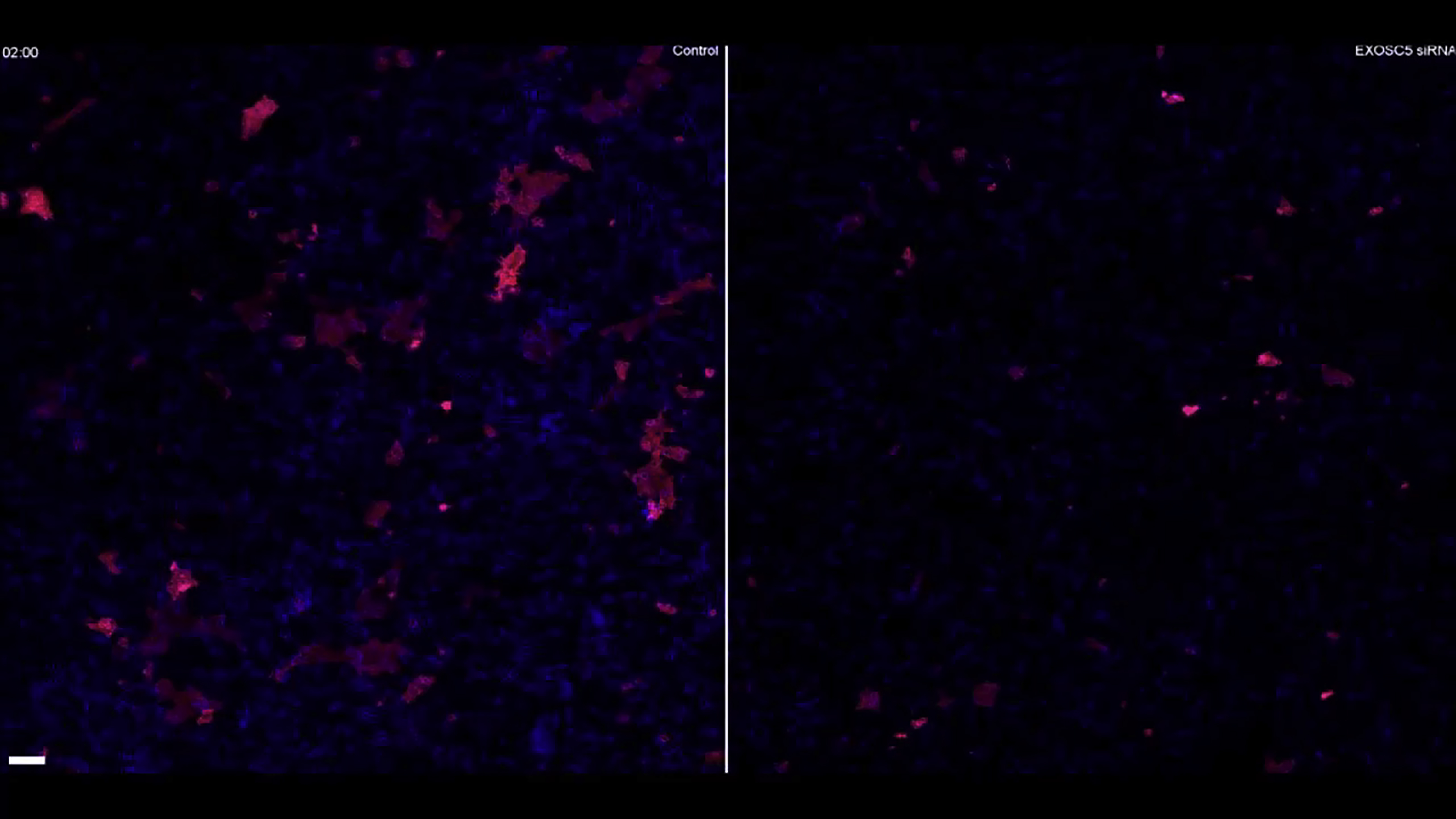

Supplement: Movie S6. Protrusions Initiate but Are Not Stable and Retract Back in Exosome Core-Depleted MDA-MB231 Cells, Related to Figure 6 — Control (left) or EXOSC5-depleted (right) MDAMB231 mKate CAAX cells were seeded on collagen-coated 3-μm transwell filters and time-lapsed for 4 hr at 30-min intervals as they formed protrusions through the pores of transwell filters. Red, cell membranes; blue, filter. Scale bar, 20 μm. The images show protrusions at the bottom of the filter. [file mmc7.jpg]

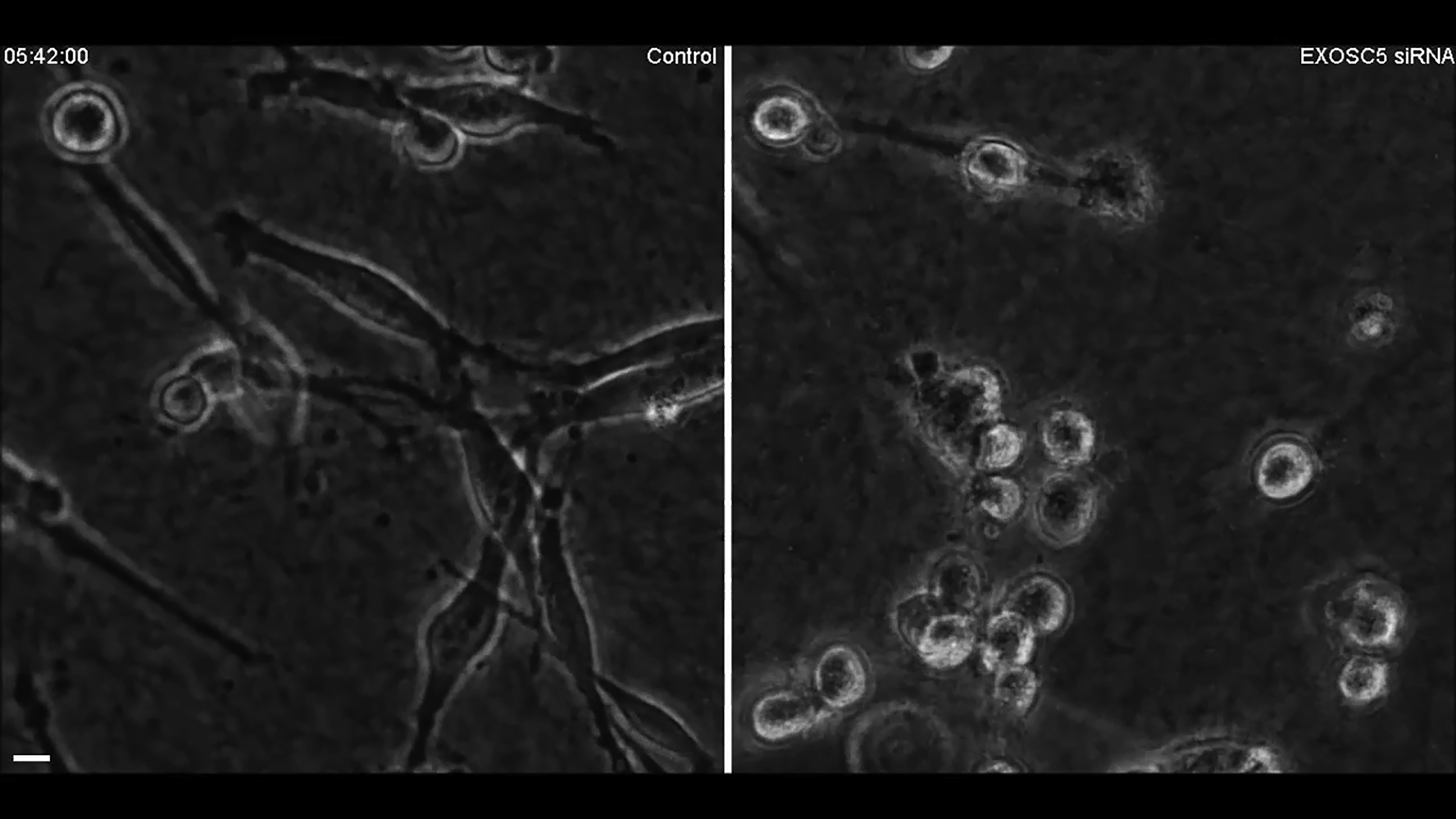

Supplement: Movie S7. Protrusions Initiate but Are Not Stable and Retract Back in Exosome Core-Depleted MDA-MB231 Cells in 3D Collagen, Related to Figure 6 — Control (left) or EXOSC5-depleted (right) MDA-MB231 cells were seeded on 3D pepsinized collagen-I gels and time-lapsed for 10 hr at 3-min intervals as they formed protrusions. Scale bar, 10 μm. [file mmc8.jpg]
